# Soil amendments reshape alpine meadow microbial communities across degradation gradients by modulating nutrient availability

**DOI:** 10.1371/journal.pone.0359576

**Published:** 2026-11-03

**Authors:** Na Wang, Zhonghua Zhang, Huakun Zhou, Yanbin Wen, Xin Wang, Huiyu Song, Yangong Du

**Affiliations:** 1 Northwest Institute of Plateau Biology, Chinese Academy of Sciences, Xining, Qinghai, China; 2 University of Chinese Academy of Sciences, Beijing, China; University of Salento: Universita del Salento, ITALY

## Abstract

Climate change and overgrazing have led to the continued degradation of alpine meadows on the Qinghai–Tibet Plateau, thereby severely impairing the ecosystem services provided by these grasslands. Soil amendments are an important strategy for restoring degraded grasslands; however, the regulatory effects of different amendment measures across varying degradation gradients and the underlying microbial response mechanisms remain unclear. This study systematically investigated the effects of three soil amendments (humic acid, superabsorbent polymer, and *Bacillus subtilis*) on soil physicochemical properties and microbial community structure in mildly, moderately, and severely degraded alpine meadows. The results indicated that the regulatory effects of soil amendments are significantly dependent on the degradation gradient: In the severe degradation stage, amendments significantly increased available nutrients, lowered pH, and alleviated alkalization, whereas their regulatory effects were limited in the mild degradation stage. Amendments selectively altered the abundance of microbial groups involved in organic matter degradation, nitrogen cycling, and stress-tolerant symbiosis. Humic acid demonstrated the optimal regulatory effect in the mild degradation stage, whereas superabsorbent polymers and *Bacillus subtilis* were more suitable for restoration in the moderate and severe degradation stages. Redundancy analysis indicated that bacterial communities were primarily subject to deterministic selection by soil pH, available potassium, and organic matter gradients, with the intensity of environmental selection increasing with increasing degradation severity; fungal communities exhibited significant environmental responses only in the moderate degradation stage, while stochastic processes dominated community assembly in the mild and severe degradation stages. In summary, alpine meadows at various stages of degradation require differentiated remediation strategies. Precise, stage-specific remediation tailored to degradation severity can facilitate synergistic restoration of the soil–microbial system, providing a theoretical basis and practical guidance for restoring degraded alpine meadows on the Qinghai–Tibet Plateau.

## 1. Introduction

Over the past half-century, the significant degradation of alpine grasslands has attracted considerable worldwide attention [1–4]. Grassland ecosystems comprise plants, microorganisms, and soils that interact in complex ways, and degradation reflects an imbalance in these systems [5]. Global climate change and prolonged overexploitation can modify plant and soil traits, thereby altering microbial diversity and community composition and exacerbating grassland degradation [6–8]. Changes in the soil environment can also affect microbial communities; for example, reduced soil water-holding capacity can alter anaerobic bacterial diversity [9,10]. Building on this perspective, the holistic restoration of coupled biotic–abiotic systems is recognized as an effective approach to mitigate grassland degradation. Empirical evidence indicates that soil amendments improve soil properties and enhance microbial activity, thereby creating a favorable environment for plant growth. Accordingly, the long-term benefits of soil amendments have led to their widespread use in restoration practices [11,12].

Humic acids (HAs), water-retaining agents, and microbial fertilizers are commonly used soil amendments because they balance utilization costs and effectiveness [13,14]. Mechanisms vary among amendment types. HA, which contains –COOH, –OH, and phenolic-OH groups, as well as quinone groups and has a high cation exchange capacity, enhances nutrient ion retention. These characteristics improve soil physicochemical properties and stimulate microbial activity [15,16]. Evidence has demonstrated that HA application increased the abundance of Firmicutes by 1.16-fold and boosted the abundances of Basidiomycota and Mortierellomycota by 81.95% and 3.16-fold, respectively [17]. Such microbial enrichment synchronously activated soil organic matter decomposition and nutrient cycling. Firmicutes promoted plant litter mineralization, Basidiomycota enhanced recalcitrant lignin degradation, and Mortierellomycota facilitated phosphorus solubilization while providing mycelial residues for stable carbon formation. Potassium-based superabsorbent polymer (K-SAP), which is rich in hydrophilic moieties, can effectively retain and gradually release water. The K-SAP application can increase the soil’s water-holding capacity by over 30%, thereby enhancing moisture conservation and reducing nutrient loss [18,19]. Microbial fertilizers, such as *Bacillus subtilis* (*B. subtilis*), can significantly improve rhizosphere microbial community diversity and enhance soil quality by directly introducing beneficial microorganisms or stimulating indigenous microbial activity [20]. Some research reported that applying *B. subtilis* reduced the abundance of the nitrogen-fixing *nifH* gene by half and upregulated the denitrification genes *narG, nirS, nirK,* and *nosZ* in soil. Replacing half of the urea application with *B. subtilis* reduced nitrogen (N) losses by 54% [21,22]. Ahmed demonstrated that microbial fertilizers increased the uptake of N, phosphorus (P), and potassium (K) by 10%–30%, with particularly pronounced effects on P and K availability [23].

However, existing soil‑amendment studies largely rely on small‑scale simulation experiments or focus only on a single degradation level [20,24–26]. Two major knowledge gaps persist. (1) Few investigations have compared soil amendment responses across multiple degradation gradients, and how restoration performance changes with degradation severity remains unclear. (2) Regional variability, as well as gradient‑specific shifts in soil microbial diversity and community composition and their linkages with soil physicochemical properties, are rarely incorporated into restoration assessments. Consequently, this knowledge gap hinders our mechanistic understanding of ecosystem responses to amendments along degradation severity. Accordingly, this study addresses these gaps by evaluating amendment effects along a full degradation gradient. This knowledge gap creates uncertainty about the pathways through which amendments act and the feedback mechanisms mediated by microbes, hinders our understanding of multifaceted soil–microbe interactions, and may hinder the selection of appropriate amendment strategy and limit our ability to fully assess improvements in soil health.

Therefore, we aimed to establish experimental plots in Qumalai County, Yushu City, Qinghai Province. HA, K-SAP, and *B. subtilis* were applied at different rates to mildly, moderately, and severely degraded alpine meadow (AM). A suite of analytical techniques was used to answer the following questions: (i) How do soil physicochemical characteristics and microbial communities respond to the AM degradation gradient and the application of amendments at different rates? (ii) How do interactions between soil properties and microbial communities change in response to each amendment, and which key factors govern microbial community structure? Addressing these questions provides a scientific basis for nature-based remediation schemes, helps identify the mechanisms underlying restoration, and establishes a framework for the targeted restoration of degraded AMs. These findings have broader implications for sustainable grassland management.

## 2. Materials and methods

### 2.1 Study sites

This study was carried out within Sanjiangyuan National Park, Qinghai Province, China. Specifically, the sampling sites are situated in the core conservation zone near National Highway 215, Qumahe Township, Qumalai County, Yushu Tibetan Autonomous Prefecture (34.9418°N, 94.6995°E). The area lies at an elevation of 4,479 m and has a subfrigid plateau climate, characterized by hypoxic conditions and frequent wind-blown sandstorms. The mean annual air temperature was −3.3 °C, and the temperature during the warm season (May–September) ranged from 3.2 to 9.4 °C. Annual sunshine duration was 2,750.2 h, with the highest duration occurring in spring at 715.5 h. Mean annual precipitation was 472.9 mm, approximately 90% of which fell from May to September, peaking at 97.6 mm in July. These climate patterns resulted in a distinct heat–rainfall synchronization during the growing season. The local vegetation belongs to alpine meadow, dominated by *Kobresia tibetica*, *Kobresia humilis*, *Potentilla anserina*, *Ajania tenuifolia*, and *Saussurea salsa*. The soil is classified as alpine meadow soil and is carbonate-type.

### 2.2 Degradation classification and experimental design

A space-for-time approach was adopted using plots along a local degradation gradient to mirror the temporal course of degradation. In February 2024, three stages of AM degradation (mild, moderate, and severe) were identified using vegetation cover, proportion of sedge and grass as the criteria (Table 1). This classification followed the Chinese national guideline GB19377−2003 and Ma et al. [27]. Given their close geographical proximity, the three sites have parent material uniformly derived from sand‑shale weathering products and exhibit carbonate‑type soil geochemical properties. Classified within the alpine meadow soil zone in soil regionalization, these sites are designated as subalpine steppe soil under genetic soil classification and Cryic Cambosols according to the Chinese Soil Taxonomy. The soil is sandy loam (sandy‑skeletal soil), with the A horizon (humus‑eluvial topsoil layer) of about 13 cm and the A/C horizon of 61 cm. They were subject to similar climate conditions.

**Table 1:** Criteria for degradation and locations of the sampling sites.

Degradation Stages	Plant coverage (%)	Bare ground coverage (%)	Sedge and grass (%)	Latitude/Longitude	Altitude/m
Mild	> 80	< 5	50–80	35°34’18"N 95°10’47"E	4,471
Moderate	50–80	5–20	30–50	35°34’20"N 95°10’01"E	4,472
Severe	< 30	> 50	< 10	35°34’19"N 95°10’59"E	4,467

Three independent sites representing three degradation stages were selected in this study. For each degradation stage site, 21 randomly selected 1 × 1 m plots (three replicate plots for each of the seven treatments) were laid out, resulting in a total of 63 experimental plots across the three degradation gradients. Based on previous studies and the characteristics of degraded alpine meadows [28–30], application rates of 5 g m^-2^ (the minimum effective dose) and 10 g m^-2^ (the economically optimal dose) were selected for this study. Treatments included HA (5 and 10 g m^-2^; HA5 and HA10), K-SAP (5 and 10 g m^-2^; K5 and K10), *B. subtilis* (5 and 10 g m^-2^; B5 and B10), and an untreated control. The materials used were as follows: analytical-grade HA containing fulvic acid and HA (Shanghai Yuanye Bio-Technology), 20–80 mesh K-SAP (Shandong Yousuo Chemical Technology), and *B. subtilis* powder (≥ 2.0 × 10^8^ CFU g^-1^, Aoston Biotechnology). The soil amendments were powdered. Top layer surface soil was collected from each plot, thoroughly mixed with the corresponding soil amendment, and then evenly spread back onto the experimental plot.

### 2.3 Soil sampling

Field sampling was conducted in June 2024. Within each plot, including control and amended plots, soil was collected using a three-point sampling method. Three soil cores were taken from the 0–10 cm soil layer using a 7 cm diameter soil auger, with sampling points located at least 10 cm from the plot edges to minimize edge effects [31]. All three soil cores from the same plot and soil layer were thoroughly combined and homogenized to form a single composite sample. The homogenized bulk soil was gently disaggregated and sieved through a 2 mm mesh to remove roots, dead fine roots, stones, and plant debris, thereby retaining the fine soil fraction [32,33]. The processed soil sample was further divided into two independent subsamples. One subsample was stored in a portable refrigerated container at 4 ℃ and transported to the laboratory within 24 h for the determination of soil physicochemical properties.. The other subsample was immediately frozen in liquid nitrogen and transported to Shanghai Haoweitai Biotechnology Co., Ltd. for shotgun metagenomic sequencing and microbial taxonomic analysis.

### 2.4 Extraction and sequencing of soil microbial DNA

Nine artificial DNA sequences with randomized nucleotide compositions and negligible identity to known natural microbial gene sequences were designed and synthesized by Genesky Biotechnologies Inc.(Shanghai, China). The nine sequences were partitioned into four 10-fold serial dilution gradientsspanning a known copy-number range. Prior to extraction, a defined aliquot of the gradient mixture was spiked into each soil sample; the exact copy number added per sample was recorded together with the soil mass processed (g), so that abundances could be back-calculated to copies per gram of soil. The spike-in corresponded to 0.1% (m/m) of the total DNA mass (see Note 1).

Total genomic DNA was extracted from soil samples using the FastDNA® SPIN Kit for Soil (MP Biomedicals, Santa Ana, CA, USA) following the manufacturer`s instructions. The mass of soilprocessed per sample was recorded. The spike-in mixture was added to the soil sample immediately after cell lysis, and extraction was then continued so that the internal standard underwent the same extraction, shearing and library-preparation losses as the sample DNA. DNA integrity was assessed by agarose gel electrophoresis, and DNA concentration was quantified using a Qubit 3.0 Spectrophotometer (Thermo Fisher Scientific, Waltham, MA, USA).

The DNA (sample plus spike-in, co-processed) was mechanically sheared using a Covaris® system (Covaris, Woburn, MA, USA) to generate fragments of 300–400 bp. Whole-genome shotgun metagenomic libraries were constructed using a ligation-dependent library preparation kit (GeneSky, Shanghai, China), including end-repair, A-tailing and adapter ligation. The 500–600 bp fraction of the sequencing library (librarylength = insert plus adapter sequences) was size-selected and recovered using MagicPure® Size SelectionDNA Beads (Transgen, Beijing, China). Library quality was assessed on an Agilent 2100 Bioanalyzer (Agilent, Santa Clara, CA, USA), and library concentration was quantified using the KAPA Library Quantification Kit (Kapa Biosystems, Wilmington, MA, USA) on an Applied Biosystems 9700HT Fast Real-Time PCR System (ThermoFisher Scientific, USA). Sequencing was performed on an Illumina NovaSeq 6000 platform (Illumina, San Diego, CA, USA) in paired-end mode (2 × 150 bp), generating approximately 10 Gb of raw data per sample.

Raw reads were quality-filtered to remove low-quality reads, adapter sequences and host-derivedsequences. Spike-in reads were identified in the filtered data and counted for each dilution level. For eachsample, a standard curve was constructed by plotting the known copy number of each gradient sequenceon the x-axis against the length-normalized read count on the y-axis, after log10 transformation of bothaxes (see Note 3): log_10_(read count / length) = m· log_10_(known copies) + bwhere “length” denotes the length (bp) of the corresponding gradient sequence. A weighted linearregression (weights = 1/x^2^) was fitted; samples with R^2^ < 0.94 were excluded or re-sequenced. Theabsolute copy number of each microbial gene in the sample was calculated by inverse prediction from itsobserved length-normalized read count using the fitted equation, and normalized to the soil mass to yieldcopies·g^−1^ soil.

Clean reads were de novo assembled using MEGAHIT (v1.x) to generate contigs; contigs ≥ 1,000 bpwere retained, and contigs ≥ 1,500 bp were used for binning with MetaBAT2. Resulting MAGs weredereplicated and assessed for completeness and contamination using CheckM / CheckM2; only bins with≥ 50% completeness and ≤ 10% contamination were retained. Gene prediction was performed on contigsusing Prodigal (-p meta). A non-redundant gene catalog was constructed by clustering gene sequences(cd-hit-est, 95% identity, 90% coverage). Gene abundance profiles were calculated by mapping cleanreads to the non-redundant gene set (Bowtie2 / minimap2, ≥ 95% identity); read counts were length-normalized (RPKM / TPM) before downstream analysis.

### 2.5 Measurement of soil physicochemical properties

Soil pH was determined using a pH meter (FE28K, Mettler-Toledo, Shanghai). Soil organic matter (SOM) was analyzed using the potassium dichromate sulfuric acid oxidation method, and available potassium (AK) was quantified using flame atomic absorption spectroscopy. Available phosphorus (AP) was extracted with 0.5 mol/L NaHCO_3_ (pH 8.5) and determined using the colorimetric method. Ammonium nitrogen (NH_4_ ⁺ –N) was assessed using the indophenol blue colorimetric assay, and nitrate nitrogen (NO_3_^-^ –N) was determined using ultraviolet spectrophotometry. All measurements followed the protocols in the Soil Agrochemical Analysis Manual.

### 2.6 Data analysis

Microbial diversity indices were calculated based on the species abundance matrix using the vegan package in R. Before analysis, soil physicochemical properties and microbial diversity data were scaled to a range of 0–1. The Shapiro–Wilk and Levene`s tests were performed to assess the normality of the distribution and the homogeneity of variances, respectively. Because the homogeneity-of-variance assumption was validated, Welch`s t-test was used to compare each treatment group with the control group. To mitigate type I error inflation arising from multiple pairwise comparisons across the groups, the significance threshold (0.05) was adjusted using the Bonferroni correction. The results are expressed as mean ± standard error (n = 3).

Linear discriminant analysis effect size (LEfSe) was used to identify microbial biomarkers that differed significantly among groups. The analysis comprised three sequential steps: (1) screening features with significant intergroup differences using the Kruskal–Wallis rank-sum test (*P* < 0.05); (2) performing pairwise comparisons using the Wilcoxon rank-sum test; (3) calculating the LDA score for each differentially abundant taxon. The analysis was performed hierarchically from the phylum to genus level using relative abundance data. Visualization was implemented using the microeco package in R, generating LDA score bar plots with column lengths proportional to LDA scores and colors indicating treatment groups and cladograms with node sizes representing relative abundance and colors indicating taxa enriched in specific treatments.

To elucidate the effects of environmental variables on microbial community structure, redundancy analysis (RDA) was performed. Species abundance data were subjected to Hellinger transformation, and environmental variables were standardized using z-score transformation. Detrended correspondence analysis (DCA) yielded gradient lengths of less than 3.0 on all axes, indicating that the community data were suitable for a linear ordination model and supporting RDA. The RDA model was constructed using the RDA function in the vegan package, and the significance of the model and individual environmental factors were assessed using 999 Monte Carlo permutation tests Variance inflation factors (VIFs) were calculated to identify variables with multicollinearity (VIF < 5); variables with VIF ≥ 5 were excluded. In the ordination biplot, the angles between environmental-variable arrows were used to approximate correlations among environmental variables. All figures were refined for publication using Adobe Illustrator CC 2018.

## 3 Results

### 3.1 Soil quality responses to amendments across degradation gradients

Firstly, grassland degradation reduced soil nutrients and increased soil pH (Table 2). Moreover, the effects of soil amendments on soil nutrients exhibited a degradation gradient dependence (Fig 1). During the mild degradation stage, soil amendments adversely affected AK and AP, but improved N availability to some extent (non-significant differences except for the HA treatment). Specifically, compared to the control group, the *B. subtilis* treatment at 10 g m^-2^ (B10) increased NH_4_ ⁺ –N and NO_3_^-^–N contents by 15.68% and 91.37%, respectively. All soil amendments significantly elevated soil pH values (*P* < 0.05). In contrast, at the moderate degradation stage, soil amendments generally increased AK and AP but reduced N availability; significant differences were observed only in B10 and K10 treatments. No significant variation was detected in soil pH, while soil organic matter content increased slightly. During the severe degradation stage, soil amendments had more pronounced regulatory effects on available nutrients. Nearly all soil amendments effectively increased concentrations of available soil nutrients (AK, AP, and NO_3_^-^–N). The HA10 treatment increased AP, AK, and NO_3_^-^–N by 24.54%, 38.95%, and 148.00%, respectively. Additionally, *B. subtilis* treatments (B5 and B10) significantly increased NO_3_^-^–N content by 168.99% and 159.80%, respectively (*P* < 0.05). Furthermore, all soil amendments reduced soil pH at the severe degradation stage.

**Table 2:** Soil chemical properties under different degradation gradients.

Soil properties factors	Mild degradation	Moderate degradation	Severe degradation
NH_4_ ⁺ –N (mg kg^–1^)	1.19 ± 0.04a	1.28 ± 0.11a	0.94 ± 0.08a
NO_3_^–^–N (mg kg^–1^)	3.87 ± 0.42a	3.63 ± 0.65a	2.38 ± 0.63a
AP (mg kg^–1^)	6.69 ± 0.23a	6.05 ± 0.25a	3.55 ± 0.15b
AK (mg kg^–1^)	82.81 ± 5.30a	73.86 ± 2.34a	45.26 ± 3.41b
SOM (mg kg^–1^)	48.17 ± 0.83a	41.38 ± 0.19b	35.28 ± 1.70c
pH	7.43 ± 0.09c	7.87 ± 0.01b	8.10 ± 0.03a

Means ± standard errors. Within the same row, different lowercase letters indicate significant differences among degradation gradients (*P* < 0.05). NH_4_ ⁺ –N, ammonium nitrogen; NO_3_^–^–N, nitrate nitrogen; AP, available phosphorus; AK, available potassium; SOM, soil organic matter.

**Fig 1:**
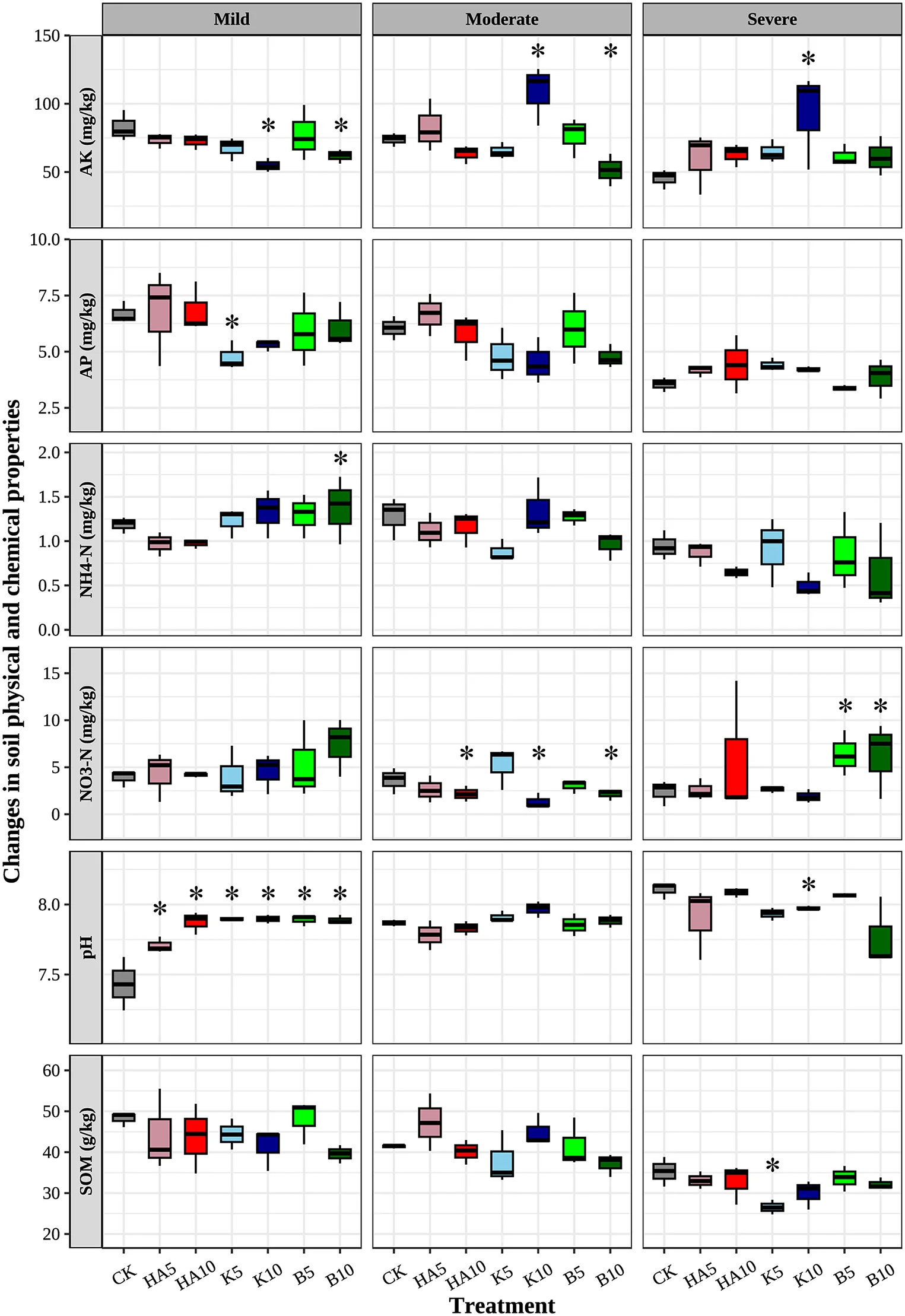
Physical and chemical properties of soil under different soil amendments. Mild, moderate, and severe represent mild, moderate, and severe stages, respectively. HA5, application of 5 g m^–2^ of HA; HA10, application of 10 g m^–2^ of HA; B5, application of 5 g m^–2^ of *B. subtilis*; B10, application of 10 g m^–2^ of *B. subtilis*; K5, 5 g m^–2^ of K-SPA; K10, application of 10 g m^–2^ of K-SAP; NH_4_ ⁺ –N, ammonium nitrogen; NO_3_^–^ –N, nitrate nitrogen; AP, available phosphorus; AK, available potassium; SOM, soil organic matter. Different lowercase letters indicate significant differences (*P* < 0.05) among different experimental treatments under the same index and degradation gradient.

### 3.2 Soil microbial α-diversity responses to amendments across degradation gradients

Bacterial diversity indices differed significantly across grassland degradation stages. Compared to the mild degradation stage, bacterial α-diversity did not vary significantly during the moderate degradation stage. In contrast, the Simpson dominance index increased significantly by 25.00% in the severe degradation stage, while the Shannon and Pielou indices decreased significantly by 0.68% and 0.69%, respectively (*P* < 0.05). These findings indicate that intensified grassland degradation significantly reduces bacterial diversity, and the bacterial community tends to shift towards a more dominant community structure. For fungi, none of the α-diversity indices showed significant differences across degradation stages. Nonetheless, fungal richness and diversity displayed a marginal increasing trend under moderate degradation (Table 3).

**Table 3:** Soil bacterial and fungal α-diversity across degradation stages.

Soil microbes	Degradation level	Observed_species	Shannon Index	Simpson Index	Pielou Index
Bacteria	Mild	27,010 ± 127a	7.39 ± 0.01a	0.0024 ± 0.0001b	0.724 ± 0a
	Moderate	26,731 ± 136a	7.41 ± 0.01a	0.0025 ± 0b	0.727 ± 0a
	Severe	27,398 ± 283a	7.34 ± 0.01b	0.0030 ± 0.0001a	0.719 ± 0b
Fungi	Mild	511 ± 21a	3.52 ± 0.09a	0.12 ± 0.01a	0.56 ± 0.01a
	Moderate	588 ± 44a	3.73 ± 0.24a	0.08 ± 0.01a	0.58 ± 0.03a
	Severe	527 ± 31a	3.27 ± 0.05a	0.11 ± 0.01a	0.52 ± 0.01a

Data in a column without shared letters indicate significant differences at *P* < 0.05. Mild, moderate, and severe represent the mild, moderate, and severe degradation stages, respectively.

Bacterial diversity responses to different soil amendments were significantly different (*P* < 0.05). During the mild degradation stage, soil amendments exerted weak effects on bacterial richness and diversity. During the moderate degradation stage, all soil amendments reduced bacterial diversity, with a significant reduction being observed in the K10 treatment. In contrast, soil amendments exhibited the strongest promoting effects on bacterial diversity at the severe degradation stage, accompanied by the greatest reduction in bacterial richness. The HA10 treatment significantly increased the Pielou evenness and Shannon diversity indices by 1.00% and 0.84%, respectively (*P* < 0.05). Additionally, the K10 treatment increased the Pielou index by 0.81%, and the K5 treatment increased the Shannon index by 0.64% (Fig 2A).

**Fig 2:**
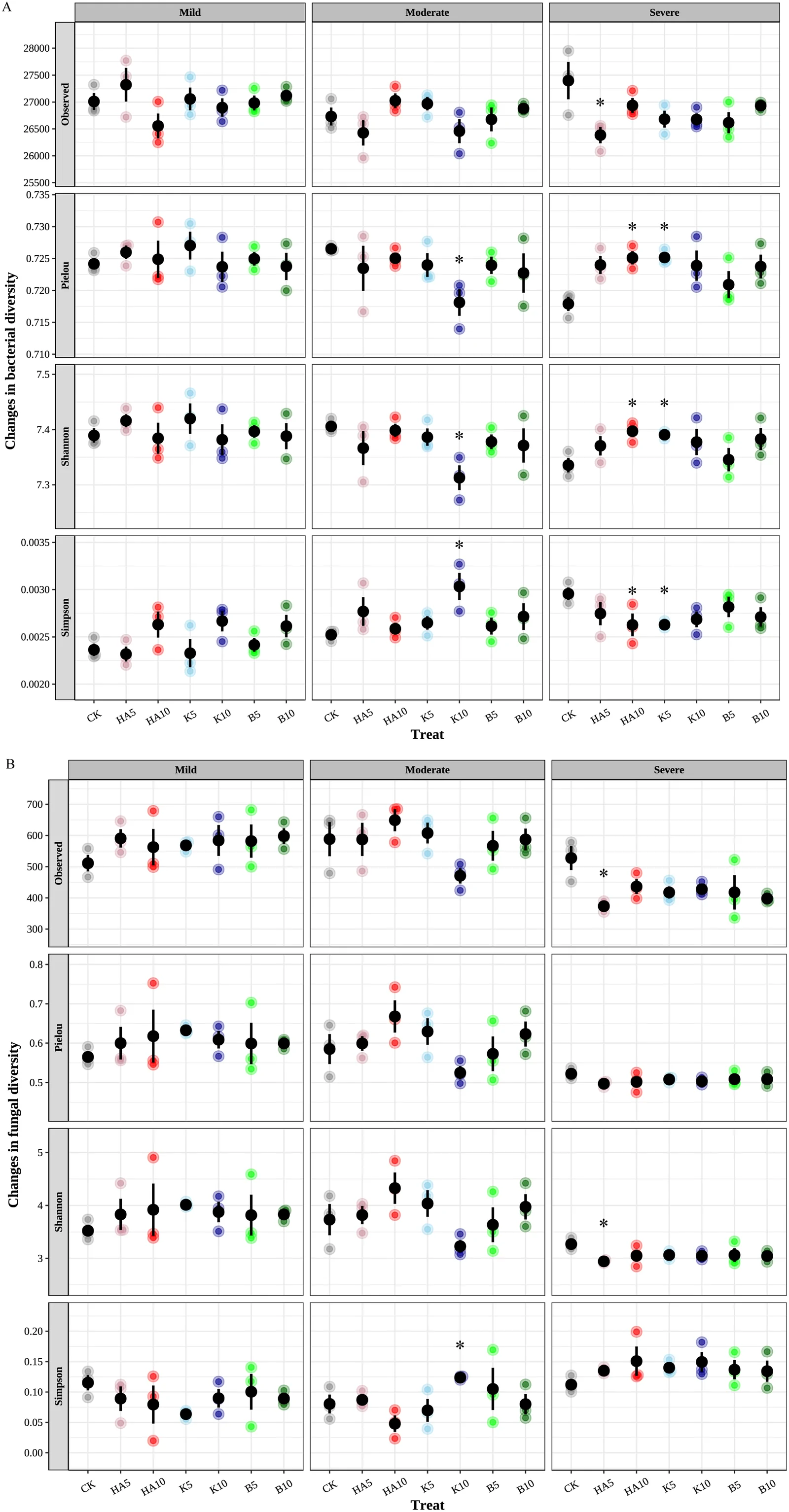
The α-diversity of soil bacterial (A) and fungal (B) communities under different soil amendments. CK, control group; HA5, the application of HA at 5 g m^–2^; HA10, the application of HA at 10 g m^–2^; K5, the application of K-SAP at 5 g m^–2^; K10, the application of K-SAP at 10 g m^–2^; B5, the application of *B. subtilis* at 5 g m ^–^ ^2^; B10, the application of *B. subtilis* at 10 g m^–2^. The asterisk denotes a significant difference in the diversity index between each treatment and the control group. * *P* < 0.05.

In contrast, fungal communities exhibited distinct response trends to soil amendments. During the mild degradation stage, soil amendments increased fungal diversity and richness, with the K5 treatment presenting the greatest promoting effect, although no significant difference was detected. Fungal community variations in the moderate degradation stage were similar to those in the mild degradation stage. HA had a more favorable influence at this stage. Specifically, HA10 treatment increased observed species, the Pielou evenness index, and the Shannon index by 10.37%, 15.14%, and 15.92%, respectively (*P* > 0.05). Meanwhile, the K10 treatment reduced fungal α-diversity indicators at the moderate degradation stage. Nevertheless, this inhibitory effect was not statistically significant. Notably, all soil amendments reduced fungal α-diversity and richness at the severe degradation stage, with the HA5 treatment producing significant inhibitory effects (Fig 2B).

### 3.3 Soil microbial community structure responses to amendments across degradation gradients

High-throughput sequencing of 63 composite soil samples yielded 4.95 × 10^12^ high-quality bacterial reads and 1.01 × 10^10^ fungal reads. Taxonomic annotation of clean reads was conducted based on a standardized gene catalog and taxonomic profiling pipeline generated 29 553 bacterial and 845 fungal operational taxonomic units. Across the soil amendments, the top bacterial phyla ranked according to relative abundance were *Pseudomonadota*, *Actinomycetota*, *Acidobacteriota*, *Bacteroidota*, *Verrucomicrobiota*, *Chloroflexota*, *Myxococcota*, *Planctomycetota*, *Gemmatimonadota*, *Cyanobacteriota*, *Candidatus Rokuibacteriota* (provisional), *Nitrospirota*, *Bacillota*, *Thermomicrobiota*, *Candidatus Tectomicrobia* (provisional), *Thermodesulfobacteriota*, *Armatimonadota*, *Ignavibacteriota*, *Bdellovibrionota,* and unclassified_Bacteria. Of these, *Pseudomonadota*, *Actinomycetota,* and *Acidobacteriota* accounted for more than 82% of the bacterial OTUs detected (S1A Fig). At the genus level (Fig 3A), the top 20 bacterial genera ranked according to relative abundance were *Sphingomonas*, *Bradyrhizobium*, *unclassified Acidobacteriota*, *Nocardioides*, *Variovorax, unclassified Betaproteobacteria*, *Mesorhizobium*, *Blastococcus*, *Solirubrobacter, Acidobacterium*, *Sphingosinicella*, *Luteitalea*, *Pyrinomonas*, *Streptomyces, unclassified Alphaproteobacteria*, *Arthrobacter, Rubrobacter*, *unclassified Chloroflexota*, and *Ramlibacter, Pseudomonas*, collectively accounting for over 40% of all bacterial genera. The genera that changed significantly after applying soil amendments were primarily those associated with organic matter degradation, such as *Sphingomonas*, *unclassified Acidobacteriota*, *Variovorax*, *Pyrinomonas*, and *Pseudomonas*.

**Fig 3:**
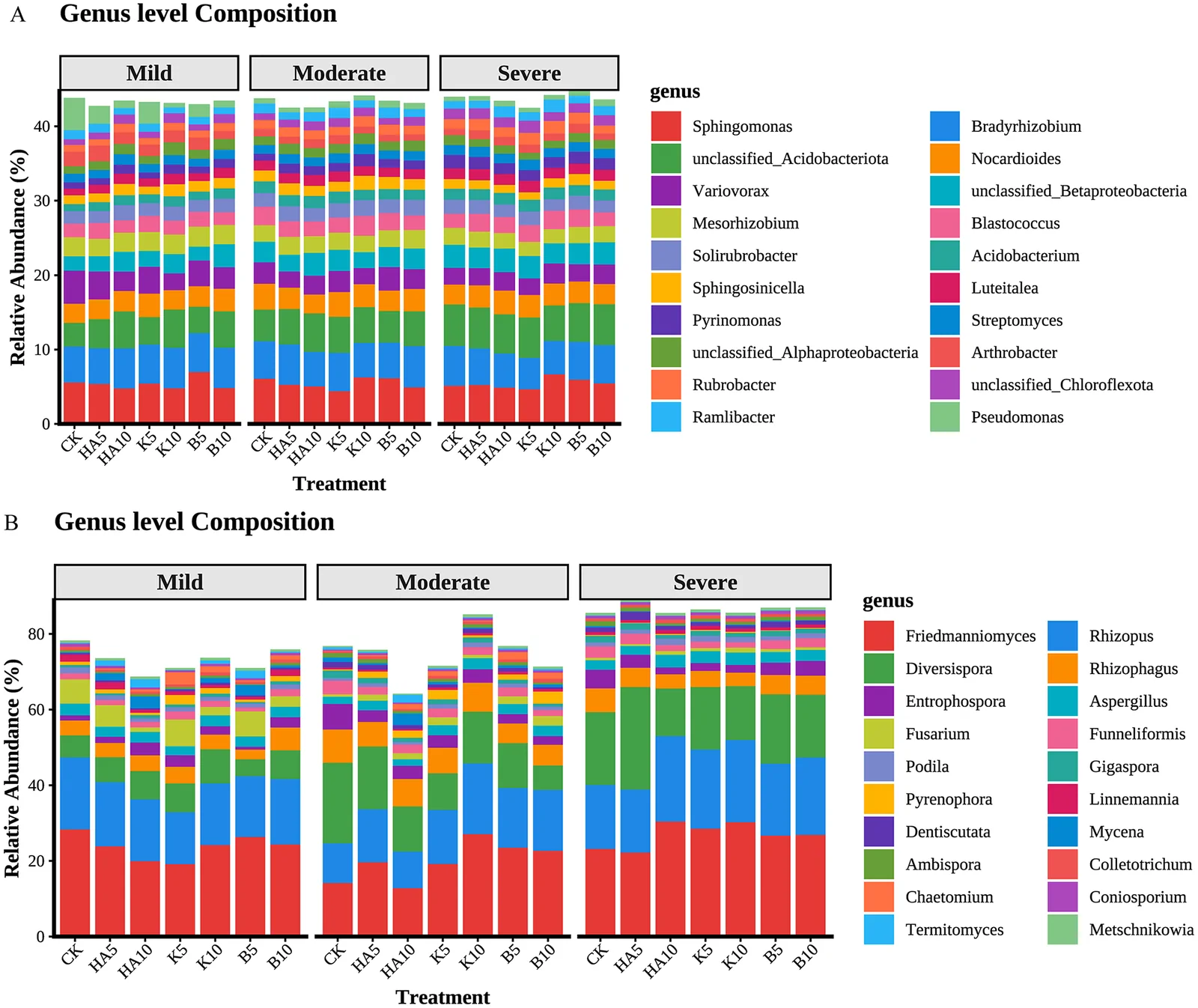
The top 20 bacterial genera ranked by relative abundance and all fungal genera under soil improvement treatments at different degradation stages. (A) The relative abundance of bacteria at the genus level. (B) The relative abundance of fungi at the genus level.

For fungal communities, the phyla ranked by relative abundance were *Mucoromycota*, *Ascomycota*, *Basidiomycota*, *Chytridiomycota*, *Zoopagomycota*, *Olpidiomycota*, *Microsporidia*, *Blastocladiomycota,* and unclassified fungi. *Mucoromycota*, *Ascomycota,* and *Basidiomycota* together represented more than 98% of total fungal OTUs (S1B Fig). At the genus level (Fig 3B), the top 20 fungal genera by relative abundance were, in order: *Friedmanniomyces, Rhizopus, Diversispora, Rhizophagus, Entrophospora, Aspergillus, Fusarium, Funneliformis, Podila, Gigaspora, Pyrenophora, Linnemannia, Dentiscutata, Mycena, Ambispora, Colletotrichum, Chaetomium, Coniosporium, Termitomyces, Metschnikowia*. These top ranked genera collectively accounted for more than 70% of the total fungal relative abundance, and exceeded 80% within the severe degradation stage.. Genera presenting significant changes after soil amendment application included *Friedmanniomyces, Diversispora, Entrophospora, Fusarium, Mycena*, and other genera associated with tolerance to extreme environments and plant symbiosis.

We screened key taxa responding to the amendment regimes at each degradation stage using multi-level LEfSe analysis (from phylum to genus). Specifically, in mildly degraded soils, this method identified 70 differentially abundant bacterial taxa and 34 differentially abundant fungal taxa significantly influenced by soil amendment type (*P* < 0.05; Figs 4A and 5A). Functional taxon analysis revealed that the dominant bacterial groups included key nitrogen cycling bacteria (*Nitrospira, Nitrosospira,* and *Bradyrhizobium*), oligotrophic adapted Acidobacteria, and Verrucomicrobia, organic-matter-degrading Proteobacteria (*Sphingomonas* and *Variovorax*), and stress-tolerant *Actinobacteria*; while the dominant fungal taxa included mycorrhizal symbiotic fungi (the ectomycorrhizal taxon *Elaphomyces granulatus* and the arbuscular-mycorrhizal family *Powellomycetaceae*), saprotrophic fungi (*Umbelopsis ramanniana* and *Penicillium cosmopolitanum)*, and biocontrol and pathogenic fungi (*Beauveria bassiana* and *Pyrenophora teres*). After soil amendment, HA10 and K10 were associated with the highest number of key bacterial groups, while HA5 and HA10 were associated with the highest number of key fungal groups (S2A and S3A Figs).

**Fig 4:**
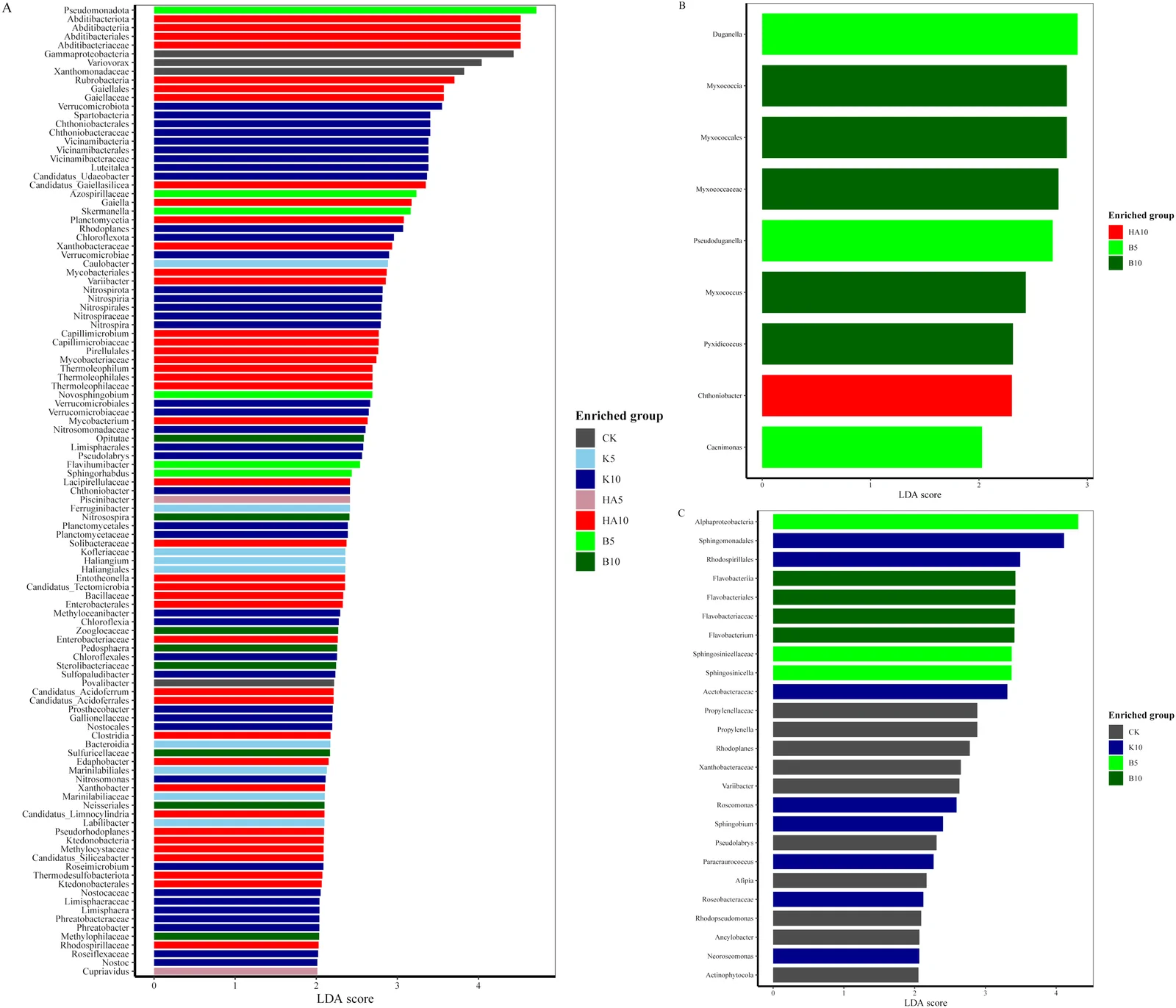
LEfSe LDA‑score bar plot of discriminative bacterial taxa under different soil amendments. Only taxa from phylum to genus are displayed; species‑level biomarkers are listed in S2 Fig. Biomarkers were identified with LDA scores > 2 and *P* < 0.05. (A) Mild degradation stage; (B) moderate degradation stage; (C) severe degradation stage.

**Fig 5:**
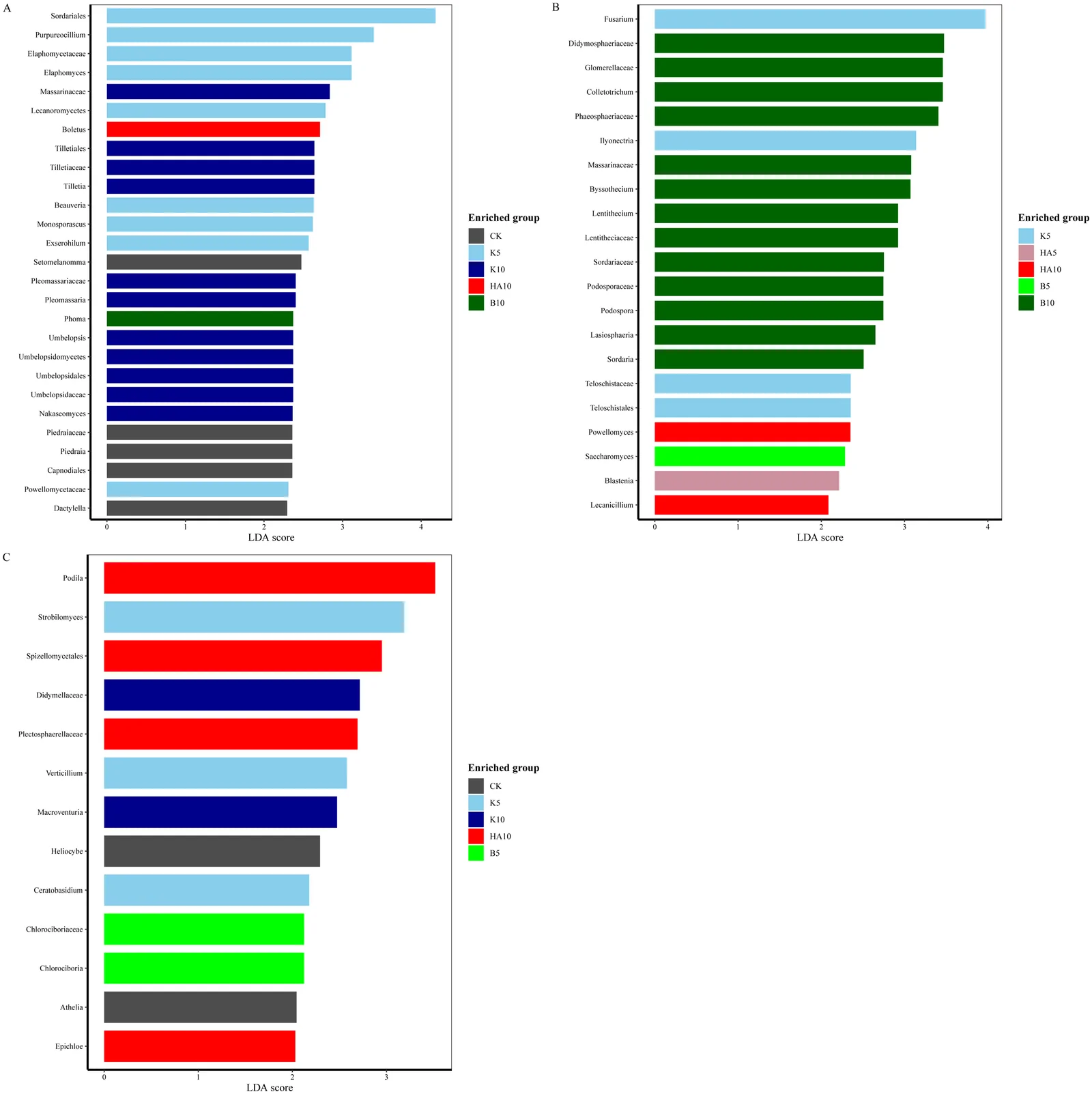
LEfSe LDA‑score bar plot of discriminative fungal taxa under different soil amendments. Only taxa from phylum to genus are displayed; species‑level biomarkers are listed in S3 Fig. Biomarkers were identified with LDA scores > 2 and *P* < 0.05. (A) Mild degradation stage; (B) moderate degradation stage; (C) severe degradation stage.

**Fig 6:**
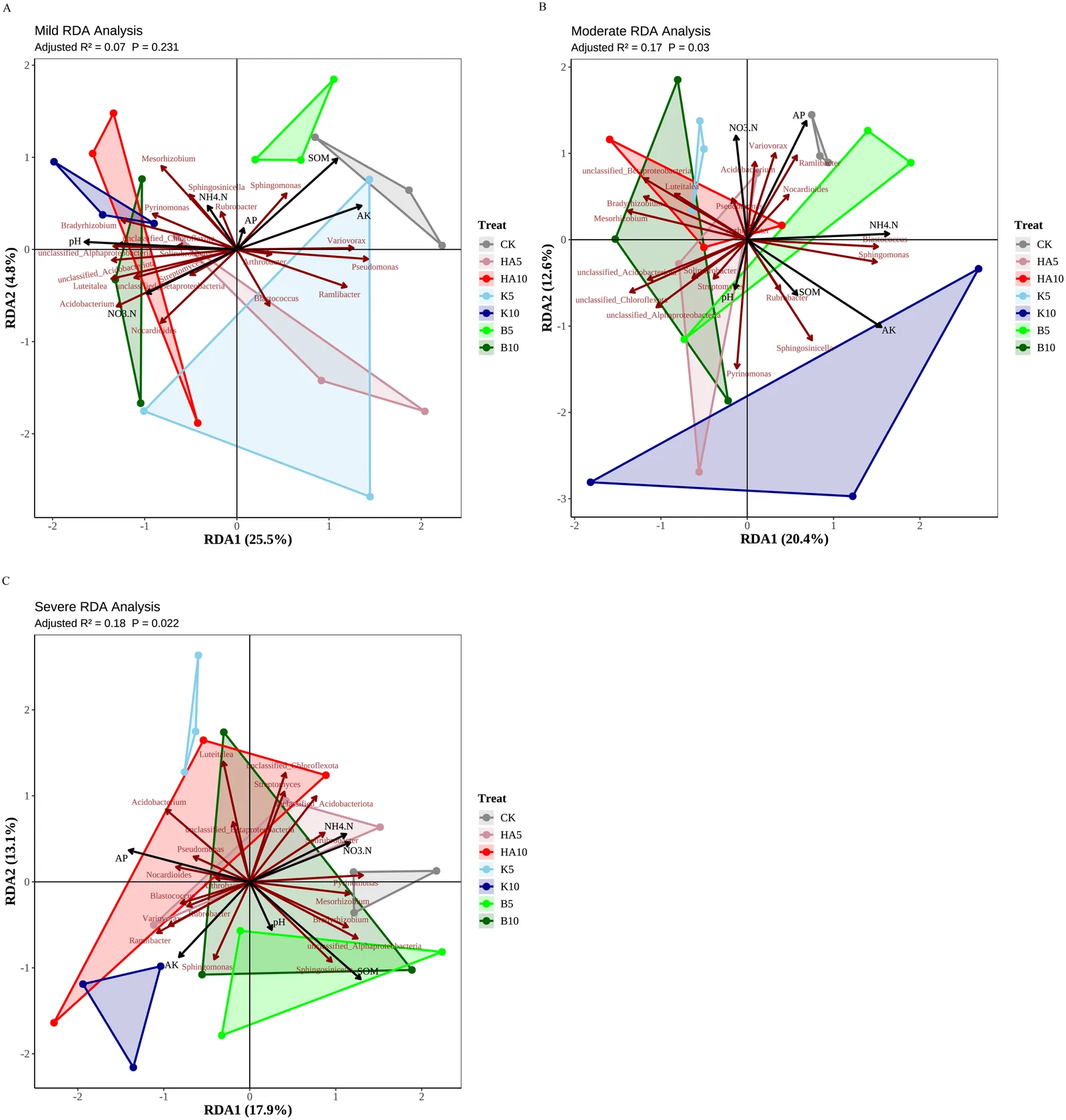
RDA of soil bacterial communities and physicochemical properties under different degradation gradients.

During the moderate degradation stage, LEfSe analysis identified 50 bacterial and 29 fungal taxa (Figs 4B and 5B). The bacterial taxa included organic matter-degrading bacteria represented by the orders Myxococcales (*Myxococcus* and *Pyxidicoccus*) and the genus *Flavobacterium* (Bacteroidota); plant-promoting bacteria (PGPR) represented by the genera *Rhizobium, Mesorhizobium*, and Massilia; pathogen-suppressing taxa represented by *Duganella* and *Nocardioides*, and soil structure-stabilizing taxa represented by *Terribacillus* (Verrucomicrobiota) and actinobacterial groups. Among fungi, saprotrophic/organic matter decomposing fungi, including the order Sordariales, the family Massarinaceae, and the genera Sordaria, Lentithecium, and Alternaria, followed by arbuscular mycorrhizal fungi, such as the family Glomeraceae, and oligotrophy-adapted fungal taxa, such as Teloschistales, Teloschistaceae, and Blastenia. In the treatment groups, B5 and B10 screened out the most important bacterial and fungal groups (S2B and S3B Figs).

During the severe degradation stage (Figs 4C and 5C), LEfSe analysis identified 41 significantly differentially abundant bacterial taxa (Pseudomonadales, Bacteroidetes, and Actinobacteria) and 21 fungal taxa (Ascomycota, Basidiomycota, and others). Functional taxon analysis further revealed that these taxa were similarly assigned to functional groups related to carbon cycling, nitrogen cycling, plant growth promotion/microecological regulation, and oligotrophic/extreme habitat adaptation. K5, K10, B5, and B10 treatments yielded the highest numbers of key microbial taxa, including fungi and bacteria (S2C and S3C Figs).

In summary, the LEfSe results indicated that different soil amendments significantly drove functional-group differentiation in bacterial and fungal communities in degraded alpine meadow soils. HA treatment significantly drove group differentiation in the mild degradation stage, while K and B treatments significantly drove group differentiation in the moderate and severe degradation stages.

### 3.4 Relationships between microbial communities and soil physicochemical properties

To investigate associations between soil bacterial community structure and environmental factors across degradation stages, we conducted RDA separately for sites with mild, moderate, and severe degradation. The results (Fig 6) revealed that the explanatory power and significance of environmental factors in explaining bacterial community variation varied across the degradation gradient. Specifically, the RDA models for moderately and severely degraded plots were both significant, with adjusted R^2^ values of 0.17 and 0.18, respectively, and *P*-values of 0.03 and 0.022, respectively. The model for mildly degraded plots did not reach statistical significance (adjusted R^2^ = 0.07, *P* = 0.231), indicating that environmental factors influenced bacterial communities during moderate and severe degradation, whereas their explanatory power is limited during mild degradation. The ordination results indicated significant differences in bacterial community differentiation patterns and key environmental drivers across different degradation stages. In the mild degradation stage, RDA1 and RDA2 explained 25.5% and 4.8% of constrained variation, respectively. Overall, the community response to the environment was weak, with only CK and B5 treatments associated with SOM. In the moderate degradation stage, RDA1 and RDA2 cumulatively explained 33.0% of constrained variation. AK and AP were the primary factors influencing the community; the K10 treatment exhibited a significant correlation with AK along the positive axis of RDA1, while CK and B5 treatments were associated with AP and NO_3_^–^–N. HA10, B10, and K5 treatments were primarily distributed along the negative axis of RDA1, associated with pH and NH_4_ ⁺ –N gradients. In the severe degradation stage, RDA1 and RDA2 cumulatively explained 31.0% of constrained variation. SOM and AP were the primary factors influencing the microbial community. Each treatment exhibited distinct spatial differentiation along the soil nutrient gradient. CK and HA5 were primarily influenced by NO_3_^–^–N and NH_4_ ⁺ –N, while B5 and B10 were closely associated with SOM. AP and AK jointly regulated HA10, K5, and K10. Associations between dominant bacterial groups and environmental factors also changed along the degradation gradients. In the mild degradation stage, species–environment associations were generally weak, with no obvious indicator groups. In the moderate degradation stage, AK and SOM gradients primarily regulated Sphingomonas and Sphingosinicella. In the severe degradation stage, bacterial groups, such as Acidobacterium and Ramlibacter, were significantly correlated with AP and AK.

The coupling patterns between fungal communities and environmental factors also changed along the degradation gradient, but their response patterns differed significantly from those of bacteria (Fig 7). Only the fungal RDA model for moderately degraded sites exhibited marginal significance (adjusted R^2^ = 0.15, *P* = 0.096 < 0.1), and the models for mildly and severely degraded sites were both insignificant, with adjusted R^2^ values of −0.04 and −0.1, respectively. This result indicates that environmental factors may drive fungal communities only under moderately degraded conditions, while their explanatory power is low during mild and severe degradation stages. Fungal ordination results revealed that under moderate degradation, RDA1 and RDA2 cumulatively explained 36.7% of the constrained variation, with the most pronounced spatial differentiation among treatment groups. SOM and AK regulated B10 and B5. HA10 was closely associated with AP, while CK and HA5 primarily responded to NH_4_ ⁺ –N and NO_3_ ^–^ –N. During the mild degradation stage, the ordination axes cumulatively explained 20.7% of the constrained variation, with a relatively uniform community structure, and only a few treatments exhibited weak correlations with SOM and AP. Samples from the severe degradation stage were scattered across the ordination space, with RDA explaining only 18.9% of the constrained variation and failing to form a stable community differentiation pattern. Overall, the association between fungal species and the environment was weaker than that between bacterial species and the environment. Chaetomium and Pyrenochaeta exhibited significant correlations with AP and SOM gradients in the moderate degradation stage, whereas fungal communities displayed weaker environmental selection in the mild and severe degradation stages, and species–environment associations were not significant. In summary, bacteria exhibited more sensitive and sustained responses to environmental factors along the degradation gradient, and moderate and severe disturbances significantly regulated bacterial community structure. In contrast, fungi exhibited sensitivity to soil physicochemical factors only during the moderate degradation stage, while under mild and severe degradation, the regulatory effect of the environment on fungal communities weakened.

**Fig 7:**
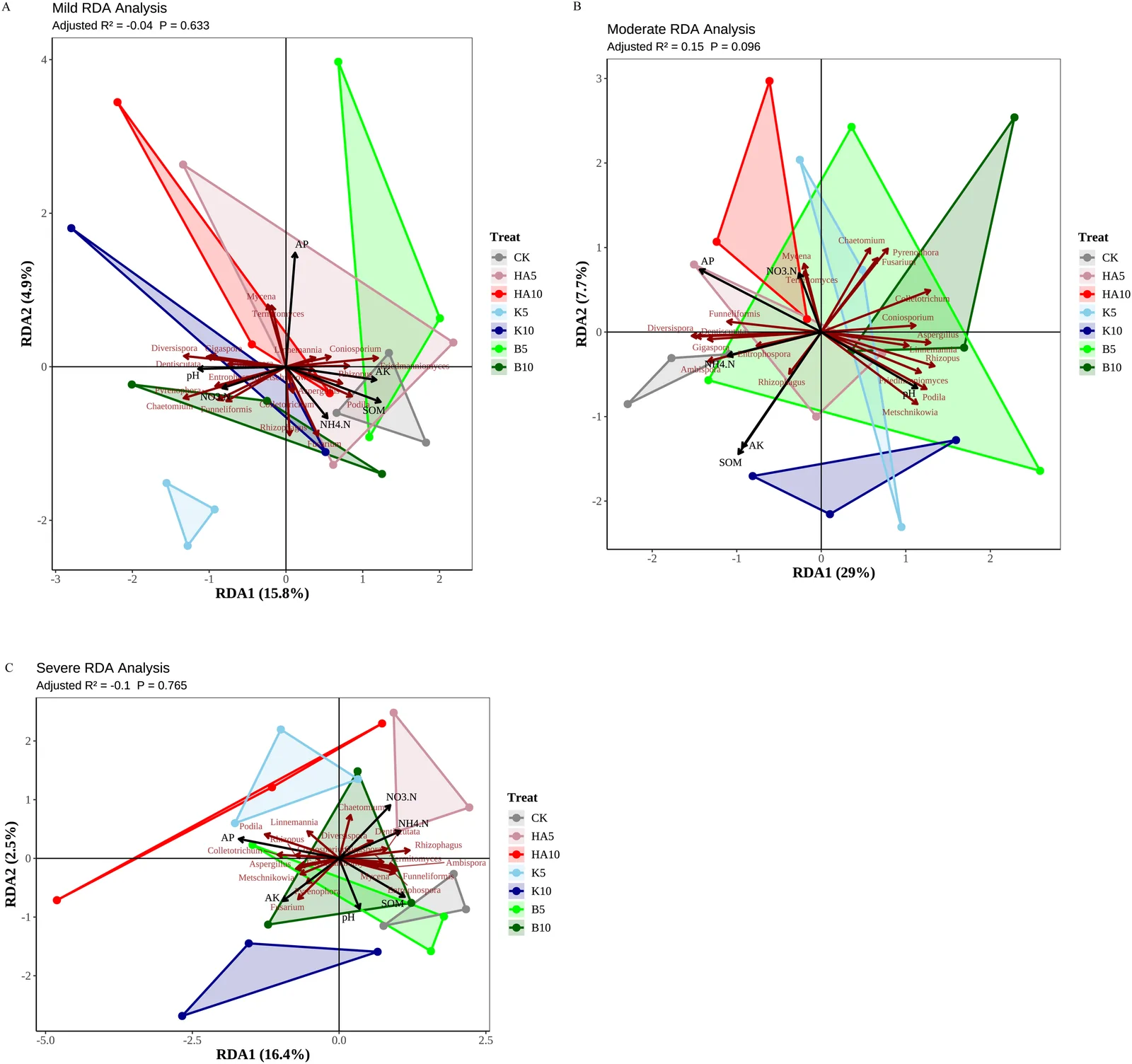
RDA of soil fungal communities and physicochemical properties under different degradation gradients.

Permutation tests based on RDA revealed significant differences in the key factors driving changes in bacterial community structure across degradation gradients (Table 4). During the mild degradation stage, soil pH was the only highly significant driving factor, explaining 17.70% of community variation (*P* < 0.01). During the moderate degradation stage, AK became a highly significant driver, explaining 15.69% of the variation (*P* < 0.01), while AP exhibited a marginally significant effect. During the severe degradation stage, SOM was a significant driver, explaining 12.76% of the variation (*P* < 0.05), while NO_3_^–^–N exhibited a marginally significant trend. The explanatory capacity of soil physicochemical variables for bacterial communities increased significantly from mild to moderate degradation and plateaued during severe degradation. Correspondingly, the proportion of residual unexplained variation declined from 64.88% in mildly degraded grasslands to 57.98% in moderately degraded grasslands and remained nearly unchanged (57.41%) under severe degradation. This pattern implies enhanced environmental filtering of bacterial communities once grassland degradation progressed to the moderate stage, with no further increase in environmental selection pressure under continuous severe degradation.

**Table 4:** Effects of physicochemical properties of soils with different degradation gradients on soil bacterial community structure.

Soil properties	Mild degradation		Moderate degradation		Severe degradation	
	Explained variance /%	*P*	Explained variance/%	*P*	Explained variance/%	*P*
pH	17.70	0.006**	2.06	0.824	3.08	0.608
SOM	4.71	0.338	7.03	0.125	12.76	0.011*
AK	1.63	0.901	15.69	0.004**	6.60	0.159
AP	3.88	0.472	8.77	0.059	7.17	0.099
NH_4_ ^+ ^ -N	3.30	0.545	4.03	0.445	4.87	0.285
NO_3_^-^-N	3.90	0.445	4.44	0.375	8.11	0.073
Residual	64.88		57.98		57.41	

Permutation tests for fungal communities indicated that their responses to environmental factors differed significantly from those of bacterial communities (Table 5). During the moderate degradation stage, soil pH had a marginally significant effect on the fungal community, explaining 10.91% of the community variation (*P* = 0.052). During the mild and severe degradation stages, all soil physicochemical factors explained little of the fungal community variation and were not statistically significant. Residual variation demonstrated that environmental factors had the highest explanatory efficiency for fungal communities at the moderate degradation stage (residual = 59.54%), while residual proportions were > 70% under mild (72.53%) and severe degradation (76.90%). This result indicates that environmental factors drive fungal communities in the moderate degradation stage, whereas fungal communities in other degradation stages were more strongly influenced by random processes or other unmeasured environmental variables.

**Table 5:** Effects of physicochemical properties of soils with different degradation gradients on soil fungal community structure.

Soil properties factors	Mild degradation		Moderate degradation		Severe degradation	
	Explained variance/%	P	Explained variance/%	P	Explained variance/%	P
pH	6.80	0.241	10.91	0.052*	1.92	0.823
SOM	5.72	0.355	8.99	0.086	5.96	0.300
AK	1.40	0.982	4.81	0.313	3.51	0.553
AP	4.67	0.515	7.20	0.169	7.95	0.211
NH_4_ ^+^ –N	7.43	0.196	3.72	0.463	1.60	0.896
NO_3_ ^-^ –N	1.45	0.972	4.83	0.324	2.16	0.782
Residual	72.53		59.54		76.90	

## 4 Discussion

### 4.1 Influence of degradation severity and soil amendments on soil physicochemical characteristics

Different soil amendments have exhibited divergent regulatory effects on soil properties [22,24,34–44]. Grasslands at different degradation levels are constrained by distinct limiting factors, making the selection of stage-specific soil amendments essential for precise alpine grassland restoration. Based on the variations in soil physicochemical properties induced by different amendment treatments across degraded alpine grasslands, this study demonstrates that targeted amendment strategies should be implemented according to grassland degradation severity.

Accordingly, amendment application produced significant shifts in soil physicochemical properties in our experiment. However, each amendment affected the degradation gradient differently. For instance, each amendment elevated soil pH under mild degradation, while pH was unchanged or even reduced under moderate or severe degradation (Fig 1). This pattern may suggests a bidirectional buffering effect: amendments exert an alkaline effect in acidic matrices, increasing pH, but exhibit an acid-buffering effect in alkaline matrices, lowering pH, collectively steering the system toward an optimal pH range. Another possibility is that increasing structural damage in highly degraded soils hampers K-SAP swelling and K⁺ exchange, thereby diminishing their ability to elevate pH [45]. Moreover, severe edaphic stress may curb the activity of HA and *B. subtilis* inoculants, preventing effective pH modulation [46,47]. AP exhibited both upward and downward shifts across treatments and stages (Fig 1), reflecting the low mobility and pronounced spatial heterogeneity of P in soils [48,49]. Amendments enhanced AK more strongly under moderate or severe degradation, plausibly because these stages suffer greater K depletion [50] and reduced CEC [51]; under such constraints, K-SAP and similar agents exchange and liberate K⁺ more efficiently.

SOM is a key factor in soil structure formation. Originating from plant/animal detritus and microbial metabolites, it is modulated by vegetation inputs and microbial turnover and immediately affects fertility, moisture-holding capacity, and erosion resistance [52,53]. None of the amendments produced a significant shift in SOM throughout the gradient. SOM declined slightly under three amendments during the mild and severe stages. Only the HA5 application elevated SOM under moderate degradation, while other treatments remained unchanged (Fig 1). We inferred that amendments enhanced aboveground biomass inputs and accelerated microbial mineralization, and the net SOM trajectory reflected the trade-off between these opposing processes.

N is indispensable for plant growth, and the pool of rapidly available N reflects the N-supply capacity of the soil [54]. NH_4_ ⁺ –N couples directly with photosynthate-derived organic acids to yield amino acids and subsequent N-rich metabolites, whereas NO_3_^–^ –N must first be reduced *in situ* to NH_4_ ⁺ –N before it can be taken up. Hence, NH_4_ ⁺ –N and NO_3_^–^–N are critical nutrient metrics [55]. These results revealed that K-SAP and *B. subtilis* increased NH_4_ ⁺ –N during mild degradation but only slightly decreased it under moderate and severe degradation, with no significant differences detected (Fig 1). The physicochemical milieu in mildly degraded soils remains favorable for ammonium retention, whereas further degradation weakens this capacity. The response of NO_3_^–^–N to amendments depended on the gradient. During mild degradation, all amendments boosted NO_3_^–^ –N. During moderate degradation, only K-SAP did so. During severe degradation, the increase was confined to HA and *B. subtilis*. This pattern reflects the textural evolution from silt to sand with increasing degradation, which enlarges porosity and curtails moisture retention [56], making K-SAP-mediated water provision crucial at the moderate stage. Conversely, severe degradation markedly depletes SOM (Table 2), and the additional carbon and enzyme inputs from HA and *B. subtilis* become dominant drivers of NO_3_^–^–N accumulation. Collectively, the gradient-dependent responses support a phased management scheme: emphasizing “moisture retention + microbial NH_4_⁺ promotion” in the early stages, and switching to “carbon enrichment + nitrate promotion” under severe degradation so that nitrogen speciation matches vegetation requirements.

### 4.2 Influence of degradation severity and soil amendments on soil microbial diversity and community structure

Soil microorganisms play pivotal roles in grassland ecosystems by regulating the decomposition of soil organic matter and plant litter and controlling nutrient availability to plants [57]. Consequently, elucidating how bacterial and fungal communities respond to degradation gradients and soil amendments is critical for understanding nutrient cycling and ecosystem function. In this study, bacterial community diversity changed significantly with grassland degradation; severe degradation significantly reduced Shannon and Pielou indices and increased the Simpson dominance index, indicating simplification and homogenization of soil bacterial communities. Although fungal α-diversity indices exhibited no statistically significant differences across degradation stages, a slight increasing trend in fungal richness and diversity was observed under moderate degradation, followed by a decline under severe degradation (Table 3). This overall trend was consistent with previous studies [58,59]. Grassland degradation lowers plant diversity, reduces litter quality and root exudate diversity, and narrows belowground resource niches. Combined with progressive soil nutrient limitations, these factors primarily drive microbial community variation across degradation stages [59–66].

These results confirmed stage-specific and divergent responses of soil bacterial and fungal communities to soil amendments along the degradation gradient, consistent with previous findings that amendment effects on microbial communities depend strongly on soil background environment and resource conditions [67–70]. The divergent microbial responses reflected distinct soil microenvironmental characteristics and microbial community adaptability at different degradation stages. During the mild degradation stage, soil nutrient status and community structure remained relatively stable. Soil amendments caused negligible fluctuations in bacterial and fungal diversity, with no significant effects of treatment, indicating that mildly degraded grassland soil had strong microecological stability and was less susceptible to disturbances from exogenous amendments [71].

During the moderate degradation stage, bacterial diversity tended to decrease under most soil amendments, yet only the K10 treatment exerted a statistically significant inhibitory effect. These results suggest that bacterial communities in moderately degraded soils are more sensitive to high-concentration potassium polymer amendment. In contrast, fungal α-diversity exhibited inconsistent trends across different amendments. Although HA10 tended to improve fungal diversity and K10 exhibited weak negative effects, all changes in fungal α-diversity induced by amendments at the moderate degradation stage were statistically nonsignificant. Accordingly, no unified directional response pattern of fungal communities to soil amendments could be identified for moderately degraded grassland. During the severe degradation stage, with severe nutrient depletion and disrupted soil structure, soil amendments effectively improved bacterial community diversity but significantly inhibited fungal α-diversity, with the HA5 treatment exhibiting the strongest inhibitory effect. This distinct discrepancy between bacteria and fungi indicates that bacteria and fungi possess divergent adaptive strategies and resource-utilization patterns under severe degradation stress [72,73].

Dose-response analysis revealed non-linear microbial responses to amendment concentrations across degradation stages. Low-dose HA and *B. subtilis* helped maintain bacterial diversity in mildly degraded soils, while high-dose amendments had stronger positive effects on bacterial diversity in severely degraded soils. Consistent with previous studies, HA application exhibited dose-dependent regulation of soil microbial community structure and function, and high-dose HA could reshape soil microbial community assembly [17,69]. As a typical plant growth-promoting rhizobacterium, *B. subtilis* significantly increased soil available N content in this study, effectively relieving nutrient limitation in severely degraded soil and providing sufficient resources for bacterial community recovery. This result supports the conclusion that high-dose *B. subtilis* amendment may be a possible option for microbial restoration in severely stressed, saline-alkaline, and degraded soils [74]. In contrast, K10 negatively affected bacterial diversity under moderate degradation. This non-linear dose response is consistent with previous reports: appropriate K-SAP addition optimizes soil water regimes, while excessive application may alter soil pore structure and resource heterogeneity, further triggering competitive exclusion of sensitive microbial taxa [67,70,75].

Soil bacterial communities in the studied degraded grassland were dominated by Pseudomonadota, Actinomycetota, and Acidobacteriota, which accounted for more than 82% of total bacterial OTU and constituted the core bacterial taxa of alpine degraded grassland soil [76]. The dominance of these oligotrophic and stress-tolerant phyla reflects the adaptive characteristics of soil microbial communities to long-term nutrient-poor conditions, with stable community composition and strong environmental resistance [77–79]. Auxiliary bacterial groups, including Chloroflexota, Myxococcota, and Nitrospirota, maintained key ecological processes, such as soil nitrogen cycling and organic matter mineralization, contributing to the functional integrity of soil microecosystems. At the genus level, Sphingomonas, Bradyrhizobium, and Acidobacterium dominated the bacterial communities. Soil amendments significantly regulated the relative abundance of genera involved in organic matter degradation and nutrient metabolism, including Sphingomonas, unclassified Acidobacteriota, Variovorax, Pyrinomonas, and Pseudomonas. These findings suggest that exogenous soil amendments can selectively regulate carbon- and nitrogen-cycling functional bacteria by improving soil nutrients and microenvironmental conditions [80].

Fungal communities exhibited stronger community aggregation than bacterial communities, with Mucoromycota, *Ascomycota*, and *Basidiomycota* occupying over 98% of total fungal sequences and forming the core of soil fungal communities in degraded grassland [81–83]. The dominant fungal genera were mainly stress-tolerant taxa and plant-symbiotic fungi, such as Friedmanniomyces, Diversispora, Entrophospora, and Fusarium. The proportion of dominant fungal genera increased to more than 80% under severe degradation, suggesting that severe environmental stress induced strong community filtering and simplified and specialized soil fungal communities [84]. Soil amendments mainly regulated the abundance of stress-tolerant, mycorrhizal-symbiotic, and pathogenic fungal genera. The relatively stable fungal community composition at the phylum level may reflect the buffering capacity of fungal mycelial networks against short-term environmental fluctuation, while genus-level symbiotic and stress-tolerant fungi were more sensitive to changes in soil physicochemical properties and nutrient conditions [85–87].

LEfSe analysis further confirmed that the regulatory effects of soil amendments on microbial functional taxa strongly depended on grassland degradation gradients, matching the habitat characteristics of each degradation stage. Mildly degraded soil had a stable microenvironment and complete ecological functions, thus harboring the highest number of amendment-sensitive differential microbial taxa (70 bacterial and 34 fungal taxa), mainly covering nitrogen cycling, organic matter decomposition, mycorrhizal symbiosis, and saprophytic functional groups. At this stage, HA and K-SAP treatments exerted the strongest regulation on microbial functional groups, confirming that HA and K-SAP could optimize microbial functional assembly and maintain the original ecological stability of mildly degraded grassland soil [51].

Moderately degraded soil was in a stressful transitional state, with moderate habitat disturbance and resource deficiency. A total of 50 bacterial and 29 fungal abundant differential taxa were identified, including organic matter-decomposing bacteria, plant growth-promoting rhizobacteria, pathogen-inhibiting bacteria, and arbuscular mycorrhizal fungi. *B. subtilis* treatments (B5 and B10) were associated with the strongest differentiation in functional taxa, indicating that microbial inoculants are more effective in restoring functional microbial groups in moderately degraded soils with organic matter loss and structural damage [49,88,89].

Severely degraded soils exhibited extreme habitat stress and severe nutrient scarcity. Correspondingly, the number of differential microbial taxa was lowest (41 bacterial and 21 fungal taxa), and the enriched key taxa were mainly stress-resistant and oligotrophy-adaptive groups involved in carbon and nitrogen cycling. The combined K-SAP and *B. subtilis* treatments significantly affected functional microbial differentiation, suggesting that amendments could effectively alleviate extreme environmental stress and select for stress-tolerant microbial communities adapted to severely degraded soil habitats [68,90].

Collectively, soil amendments regulated soil microbial communities in a stage-dependent manner by modifying soil physicochemical properties. Stage-specific strategies should be adopted for grassland restoration: high-dose HA and K-SAP benefit microbial functional optimization in mildly degraded soils; microbial inoculants exhibit stronger regulatory effects under moderate degradation; and the combined application of K-SAP and B. subtilis was most effective for microbial community reconstruction in severely degraded alpine grassland soil.

### 4.3 Relationship between microbial community composition and soil physicochemical attributes across degradation gradients in response to soil amendments

The results indicate that bacterial and fungal communities respond differently to soil physicochemical factors, and that both the intensity of environmental selection and the key driving factors vary among degradation stages, depending on the degree of soil degradation.

Environmental dependence of bacterial communities increased from mild to moderate degradation and plateaued under severe degradation. In the moderate and severe degradation stages, the overall RDA models were significant, and environmental factors explained spatial variation in bacterial communities, whereas in the mild degradation stage, environmental factors were less strongly associated with community structure. The physicochemical properties of soils in mildly degraded grasslands are relatively stable, and habitat stress is low; only soil pH significantly shaped bacterial community composition, serving as the primary environmental filtering factor at this stage (explanatory power 17.70%, P < 0.01). As degradation progressed to moderate levels, the soil nutrient pattern restructured, with AK becoming the dominant factor driving bacterial community differentiation (explanatory power 15.69%, P < 0.01). AP also exhibited a potential regulatory role, and communities among amendments exhibited significant spatial segregation along nutrient gradients. The K10 treatment was closely associated with the AK gradient along the positive RDA1 axis, indicating that it reshaped bacterial community composition by altering soil AK. By the severe degradation stage, soil organic matter loss intensified, and SOM emerged as a key limiting factor (explanatory power 12.76%, P < 0.05). N, P, and K jointly shaped the selection and differentiation of bacterial communities, and HA10 exhibited strong associations with SOM, potentially restructuring bacterial assemblages. Correspondingly, residual variance in RDA models declined sharply from mild to moderate degradation (64.88% to 57.98%) and only slightly further decreased under severe degradation (57.41%). This pattern suggests that deterministic environmental selection becomes stronger once grassland degradation reaches the moderate stage, with no further increase in environmental selection pressure under severe degradation. Correlations between soil physicochemical gradients and bacterial taxa imply that environmental filtering may favor the enrichment of stress-tolerant groups, such as Sphingomonas, Acidobacterium, and Ramlibacter, while constraining sensitive taxa, potentially shaping nutrient-dependent bacterial community assembly along the degradation gradient [91–93].

Compared to bacterial communities, the fungal communities in this study were generally less constrained by environmental factors, exhibiting weak, non-significant environmental responses at the moderate degradation stage (adjusted R^2^ = 0.15, *P* = 0.096); none of the physicochemical factors significantly explained the variation in the fungal community at the mild or severe degradation stages. Moderately degraded habitats represent a transitional disturbance stage, in which moderate fluctuations in soil physicochemical conditions induce moderate environmental filtering [94], with pH, SOM, and P and K availability jointly mediating fungal community differentiation. This promoted strong coupling between taxa, such as *Chaetomium* and *Pyrenochaeta*, and environmental gradients. Treatment B10 displayed the most pronounced regulatory effect, exhibiting a high degree of coupling with the SOM gradient. In the mild degradation stage, habitat heterogeneity was low, and selective pressure was minimal. Under severe degradation, fungi may rely on stress-resistant traits, symbiotic interactions, and functional redundancy to cope with harsh soil conditions, reducing their sensitivity to individual soil physicochemical variables. A high proportion of residual variance is common in both mildly and severely degraded fungal communities, suggesting that stochastic processes, interspecific interactions, and dispersal constraints play a dominant role in community assembly, consistent with previous studies [95]. This mechanism helps explain why fungal community α-diversity displayed no significant differences across degradation stages in the present study.

Across degradation stages, amendments shifted microbial groups by manipulating different soil nutrients, underscoring strong environmental filtering in degraded grassland ecosystems. This finding implies that targeted regulation of key variables (for example, boosting SOM with B10 or fine-tuning AK and pH with HA5) can reassemble soil microbial communities. Therefore, restoration strategies should match soil amendments to stage-specific environmental drivers, increase SOM, or adjust pH during severe degradation to facilitate the recovery of beneficial microbial guilds.

This study was based on a single‑growing‑season field experiment, and we cannot fully capture potential inter‑annual variation in soil microbial responses to amendments. Furthermore, our investigation focused primarily on short‑term changes in soil physicochemical properties and taxonomic composition; future long‑term monitoring combined with functional omics is needed to reveal the sustained functional evolution of soil‑microbe systems under restoration.

## 5. Conclusions

This study demonstrates that grassland degradation substantially depletes soil organic matter and available nutrients while elevating soil pH, which reduces bacterial diversity and evenness, whereas fungal communities show greater stability against degradation induced disturbance. Across the degradation gradient, soil amendments exerted context dependent effects on soil physicochemical properties, producing the strongest nutrient enriching and pH reducing performance under severe degradation. Bacterial diversity benefited more from amendments at higher degradation severity, while fungal communities displayed the reverse response; amendments altered the abundance of microbial taxa mediating organic matter decomposition, nitrogen cycling, and plant associated stress tolerant symbiosis. LEfSe further revealed that HA drove the greatest microbial taxon differentiation in mildly degraded grasslands, whereas *Bacillus subtilis* based inoculants and water retaining agents shaped community divergence in moderately and severely degraded sites. By modifying soil nutrients and pH, these amendments reshaped the key physicochemical drivers governing microbial community assembly. Accordingly, differentiated restoration strategies are proposed for alpine meadows at distinct degradation stages: humic acid for mild degradation, microbial inoculants for moderate degradation, and the combined application of microbial inoculants, K-SAP and high dose HA for severe degradation. Given the limitations of short-term observations from a single growing season, future long-term field studies incorporating multiomics frameworks (e.g., metagenomics and metabolomics) are required to decipher the molecular mechanisms underlying microbially mediated carbon nitrogen phosphorus cycling, which will advance theoretical understanding and deliver practical guidance for sustainable restoration of degraded alpine grasslands.

## Supporting information

S1 Fig Relative abundance of microbial phyla. The top 20 bacterial phyla ranked by relative abundance and all fungal phyla under soil improvement treatments at different degradation stages. (A) Relative abundance of bacteria at the phylum level; (B) relative abundance of fungi at the phylum level.
(DOCX)

S2 Fig Bacterial LEfSe cladogram and LDA-effect-size bar plots. Cladogram and LDA-effect-size bar plots of bacterial taxa across different degradation gradients (A: LEfSe cladogram for mild degradation; B: LEfSe cladogram for moderate degradation; C: LEfSe cladogram for severe degradation; D: LDA-effect-size bar plot for mild degradation; E: LDA-effect-size bar plot for moderate degradation; F: LDA-effect-size bar plot for severe degradation). Different colors denote different treatments.
(DOCX)

S3 Fig Fungal LEfSe cladogram and LDA-effect-size bar plots. Cladogram and LDA-effect-size bar plots of fungal taxa across different degradation gradients (A: LEfSe cladogram for mild degradation; B: LEfSe cladogram for moderate degradation; C: LEfSe cladogram for severe degradation; D: LDA-effect-size bar plot for mild degradation; E: LDA-effect-size bar plot for moderate degradation; F: LDA-effect-size bar plot for severe degradation). Different colors denote different treatments.
(DOCX)
