# Supplementary material for: Soil amendments reshape alpine meadow microbial communities across degradation gradients by modulating nutrient availability
Source: PLoS One. 2026 Nov 3;21(11):e0359576. doi: 10.1371/journal.pone.0359576 (PMC13730322; doi:10.1371/journal.pone.0359576)
Supplement: Supplementary file 1 [file pone.0359576.s001.docx]

S1. The top 20 bacterial phylum ranked by relative abundance and all fungal phylum under soil improvement treatments at different degradation stages. (A). The relative abundance of bacteria at the phylum level. (B). The relative abundance of fungi at the phylum level.
