# Supplementary material for: Soil amendments reshape alpine meadow microbial communities across degradation gradients by modulating nutrient availability
Source: PLoS One. 2026 Nov 3;21(11):e0359576. doi: 10.1371/journal.pone.0359576 (PMC13730322; doi:10.1371/journal.pone.0359576)
Supplement: Supplementary file 3 [file pone.0359576.s003.docx]

B

A

C

E

D

F

S3. Cladogram and LDA-effect-size bar plots of fungal taxa across different degradation gradients (A: LEfSe cladogram for mild degradation; B: LEfSe cladogram for moderate degradation; C: LEfSe cladogram for severe degradation; D: LDA-effect-size bar plot for mild degradation; E: LDA-effect-size bar plot for moderate degradation; F: LDA-effect-size bar plot for severe degradation). Different colors denote different treatments.
