# Supplementary material for: Soil amendments reshape alpine meadow microbial communities across degradation gradients by modulating nutrient availability
Source: PLoS One. 2026 Nov 3;21(11):e0359576. doi: 10.1371/journal.pone.0359576 (PMC13730322; doi:10.1371/journal.pone.0359576)
Supplement: Supplementary file 5 [file pone.0359576.s006.docx]

**Response to Reviewers**

**Manuscript ID: PONE-D-25-55807R1**

Dear Dr. Cardinale and Reviewer #3,

We sincerely thank you for your careful evaluation of our manuscript and for the constructive comments. We have carefully revised the manuscript to address each point raised. Below, we respond to every comment point by point. Reviewer comments are reproduced in italics, followed by our responses. All revisions in the manuscript are highlighted with tracked changes.

**Minor Criticisms**

**Previously flagged, as yet uncorrected**

**Comment 1.** *Species name still un-italicized in l. 33, 42, 80, etc.*

Response: We thank the reviewer for catching this. All Latin binomial species names (e.g., Bacillus subtilis, B. subtilis, Kobresia tibetica) and gene symbols throughout the manuscript have been checked and formatted in italics, including the passages previously noted (around ll. 33, 42, and 80) and elsewhere in the text. Phylum-level names, which do not require italicization under the International Code of Nomenclature of Prokaryotes, remain in regular font.

**Comment 2.** *Words within a line of text are broken by hyphens in line 131 and elsewhere throughout.*

Response: The manuscript has been professionally language-polished and re-typeset for this revision. All artificial line-break hyphenations (e.g., words split across lines by hard hyphens) have been removed, and the text now flows naturally without such breaks.

**Comment 3.** *Many minor typos around punctuation, e.g. at ll. 201–206; many nonstandard punctuation characters throughout.*

Response: We apologize for these errors. The entire manuscript has undergone thorough proofreading and professional English polishing. Punctuation has been standardized throughout, including em dashes, en dashes, commas, semicolons, and special characters (e.g., proper minus signs, multiplication signs, and superscript units). The specific issues around ll. 201–206 and all other nonstandard punctuation characters have been corrected.

**Comment 4.** *Fig. 1 abbreviations changed as suggested but color-coding is unchanged, in conflict with what the authors state in their response letter.*

Response: We apologize for this discrepancy in the previous round. In this revision, the color schemes of all figures—including Figure 1—have been genuinely updated. We have adopted a consistent palette with clearly distinguishable colors across treatment groups throughout Figures 1–8. The color assignments are now internally consistent across all figures and match the descriptions in the figure legends.

**New**

**Comment 5.** *ll. 151–153: New information about amendment application is helpful but should be in the past tense (describing what was done) rather than the imperative case (telling the reader what to do).*

Response: We thank the reviewer for this suggestion. The amendment application procedure has been rewritten entirely in the past tense.

**Comment 6.** *Table 3 reports observed species count to the hundredths place. The last two significant digits reported are not meaningful.*

Response: We agree. The observed species counts in Table 3 are now reported as integers with standard errors rounded to whole numbers, removing the meaningless decimal places.

**Comment 7.** *ll. 579–581: The authors write "In contrast, fungal hyphal networks possess advantages in resource acquisition and niche modification, driving the expansion of fungal communities." It's not clear on what basis they argue that fungal communities are expanding after treatment of mildly degraded soils; the differences in fungal richness reported in fig. 3B are not significant. I'm also not sure in what sense the authors mean "expanding": are they referring to total fungal abundance, or to richness, or some other measure?*

Response: We thank the reviewer for identifying this unsupported statement. The sentence claiming that fungal communities were "expanding" has been removed. In the revised Discussion (Section 4.2), we now explicitly state that "all changes in fungal α-diversity induced by amendments at the moderate degradation stage were statistically non-significant. Accordingly, no unified directional response pattern of fungal communities to soil amendments could be identified for moderately degraded grassland." For the mild degradation stage, we similarly note that "soil amendments increased fungal diversity and richness, with the K5 treatment presenting the greatest promoting effect, although no significant difference was detected" (Results, Section 3.2). We no longer infer "expansion" (in abundance, richness, or any other measure) from non-significant richness trends.

**Comment 8.** *ll. 689–690: The authors conclude section 4.2 with the strong claim that "for severely degraded soils, synergistic use of K-SAP treatment and microbial amendments is required." While I see that these treatments taken individually evoked distinct responses across the microbial groups present, this claim is completely unjustified because the authors did not in fact test the combined treatment they say is required.*

Response: We agree that the original claim overstated what our experiment could support, as no factorial combination of K-SAP and microbial inoculants was directly tested. We have revised this statement accordingly. The prescriptive wording "is required" has been removed.

**Major Criticisms**

**Previously flagged, as yet uncorrected**

**Comment 9.** *Section 3.2 of results continues to highlight results that are not statistically significant. In some cases this is flagged, though not in all (e.g. for fungi, the effects of K10 treatment in moderately degraded soils).*

Response: We have carefully revised Section 3.2 to ensure that every treatment comparison is explicitly labeled as statistically significant or non-significant.

**Comment 10.** *The LEfSe trees (figs. 5, 6) have been revised somewhat but remain quite difficult to read.*

Response: We have substantially redesigned Figures 5 and 6 to improve readability. The circular cladograms have been replaced with horizontal LDA score bar plots, which present the discriminative taxa in a clear, ranked format. To reduce visual clutter, only taxa from phylum to genus level are displayed in the main figures; species-level biomarkers have been moved to Supplementary Figures S2 (bacteria) and S3 (fungi).

**Comment 11.** *The data in table 4 do not appear to be consistent with the statement (ll. 479–481) that "overall, as the degree of degradation increased, the explanatory power of environmental factors on the bacterial community gradually strengthened".*

Response: We thank the reviewer for catching this inconsistency. The original statement was imprecise and has been corrected to accurately reflect the data in Table 4.

**Comment 12.** *ll. 581–588: although the presentation of alpha diversity data in the revised results section has become more careful about distinguishing between significant and non-significant results, the authors continue to rest their interpretation on non-significant results, as they do in this passage: e.g., "During the moderately degraded stage, all amendment treatments reduced bacterial diversity, with the inhibitory effect of the K10 treatment being significant", followed by a sentence of speculation about the purported pattern seen across treatments. Similarly for fungi in the next sentence: one treatment of moderately degraded soil gave a significant result by one measure, but the authors speculate as though the results were significant across the board.*

Response: We have revised the Discussion (Section 4.2) to eliminate speculation based on non-significant results.

**Comment 13.** *ll. 745–747: Further to the previous point, the authors argue that the differing effects of different amendments "impl[y] that targeted regulation of key variables (for example, boosting SOM with B10 or fine-tuning AK and pH with HA5) can thus re-assemble the microbial network." While this could be true, and would be terrific to establish firmly, the work presented is certainly not sufficient to support this inference, given the complex and often non-linear emergent dynamics of such systems. In particular, judging whether the microbial network has been "re-assembled" demands knowledge of the community baseline prior to disturbance.*

Response: We agree that the original claim of "re-assembling the microbial network" was too strong given the data and the absence of pre-disturbance baseline data. We have made the following revisions: (1) The phrase "re-assemble the microbial network" has been changed to "reassemble soil microbial communities," which is a less specific claim more closely aligned with our taxonomic composition data (Section 4.3). (2) The statement is now explicitly framed as an implication rather than a demonstrated conclusion: "This finding implies that targeted regulation of key variables … can reassemble soil microbial communities." (3) We have added a limitations paragraph acknowledging that "this study was based on a single-growing-season field experiment, and we cannot fully capture potential inter-annual variation in soil microbial responses to amendments," and that "future long-term monitoring combined with functional omics is needed to reveal the sustained functional evolution of soil–microbe systems under restoration" (Section 4.3; Conclusions). We agree that definitive demonstration of community (re)assembly would require pre-disturbance baseline data and long-term observation, and we have cited this as an important future direction.

**New**

**Comment 14.** *ll. 184–96: New methods details are presented in a series of sentence fragments and raise a number of new questions. First, the authors state clearly in their response letter that their "analyses are based on ASV-level datasets", but their methods do not describe amplification and do appear to describe shotgun metagenomics. (Later in the response letter, however, the authors appear to contradict themselves by saying "We explicitly state that the functional community analysis in this study was based on metagenomic sequencing data, rather than 16S/ITS amplicon sequencing"—so I am once again confused. If the authors have shotgun metagenomes, I encourage them to make use of them, to do a more detailed genome-centric analysis of the community members that respond to treatment. Such an analysis would be outside the scope of this work, but there is much more to be learned from whole metagenomes than just the taxonomic distribution of the community.) Second, statements such as "selected an appropriate assembly software" are insufficient, and the authors make no mention at all of binning nor of how they defined OTUs. Third, this paragraph is in a section about DNA sequencing but appears (e.g. ll. 188–191, "Aligned the Clean Reads to the contigs using Bowtie2, counted the number of reads mapped to each contig, and then calculated TPM values with R software. Based on the predicted contig/gene relationships, obtained the expression levels of individual genes") to be describing metatranscriptomic mapping and differential expression analysis. If indeed this is about metatranscriptomics, then we need additional details on RNA extraction, rRNA removal (if any), etc., as the extraction and library prep details in the paragraph above just cover DNA.*

Response: We apologize for the confusion caused by our previous response and by the fragmented methods text. We now clarify unambiguously: this study employed shotgun metagenomic sequencing (not 16S/ITS amplicon sequencing and not metatranscriptomics). The Methods section (Section 2.4) has been completely rewritten as coherent paragraphs and now provides full methodological detail.

We appreciate the reviewer's encouraging suggestion to perform genome-centric analysis of the metagenomes. We fully agree that MAG-resolved analysis would deepen the biological insights, and we have added this as a stated future direction in the Conclusions ("future long-term field studies incorporating multi-omics frameworks"). Such an analysis is beyond the scope of the present study, which focuses on taxonomic community responses and their environmental drivers.

**Summary of Additional Improvements**

In addition to the point-by-point revisions above, we have:

- Had the entire manuscript professionally edited for English language, grammar, and style, addressing the reviewer's assessment that the manuscript did not meet PLOS ONE's standard for intelligible, standard English.
- Ensured consistent and accurate reporting of statistical significance throughout the Results and Discussion sections.
- Corrected the RDA explanatory-power narrative to match the data in Tables 4 and 5.
- Tempered over-strong conclusions regarding combined treatments and microbial network reassembly, and added explicit study limitations and future directions.
- Clarified all sequencing and bioinformatics methods with specific software names, versions, and parameters.
- Redesigned Figures 5 and 6 as LDA bar plots with species-level taxa moved to supplementary materials, and simplified Figures 7 and 8 to genus-level labels only.

We hope that these revisions satisfactorily address all of the reviewer's concerns and that the manuscript is now suitable for publication in PLOS ONE. We thank the reviewer again for the thorough and constructive feedback, which has significantly improved the quality of our work.

Sincerely,

Na Wang and co-authors

On behalf of all authors
