# Supplementary material for: Soil amendments reshape alpine meadow microbial communities across degradation gradients by modulating nutrient availability
Source: PLoS One. 2026 Nov 3;21(11):e0359576. doi: 10.1371/journal.pone.0359576 (PMC13730322; doi:10.1371/journal.pone.0359576)
Supplement: Supplementary file 4 [file pone.0359576.s005.docx]

Journal Requirements:

When submitting your revision, we need you to address these additional requirements.

1. Please ensure that your manuscript meets PLOS ONE's style requirements, including those for file naming. The PLOS ONE style templates can be found at https://journals.plos.org/plosone/s/file?id=wjVg/PLOSOne_formatting_sample_main_body.pdf and https://journals.plos.org/plosone/s/file?id=ba62/PLOSOne_formatting_sample_title_authors_affiliations.pdf

Response:

We have carefully checked and fully followed the official PLOS ONE style guidelines and formatting requirements in the revised manuscript. We have strictly referenced the provided PLOS ONE formatting templates to standardize the overall layout, text structure, table and figure presentation, as well as file naming conventions. All format-related issues have been fully revised and unified to comply with the journal’s specifications.

2. In your Methods section, please provide additional information regarding the permits you obtained for the work. Please ensure you have included the full name of the authority that approved the field site access and, if no permits were required, a brief statement explaining why.

Response：

The field sampling sites in this study belong to public open management areas, and no specific authorization or official permit is required for field access, vegetation investigation and soil sample collection. No protected areas, private land or restricted administrative regions were involved in our field work. Therefore, no additional access permit from administrative authority was needed for the present study.

3. When completing the data availability statement of the submission form, you indicated that you will make your data available on acceptance. We strongly recommend all authors decide on a data sharing plan before acceptance, as the process can be lengthy and hold up publication timelines. Please note that, though access restrictions are acceptable now, your entire data will need to be made freely accessible if your manuscript is accepted for publication. This policy applies to all data except where public deposition would breach compliance with the protocol approved by your research ethics board. If you are unable to adhere to our open data policy, please kindly revise your statement to explain your reasoning and we will seek the editor's input on an exemption. Please be assured that, once you have provided your new statement, the assessment of your exemption will not hold up the peer review process.

Response:

We fully understand and comply with the PLOS ONE open data policy. To ensure immediate and full data accessibility, we have already provided the permanent and public data sharing link within the revised manuscript and relevant submission materials.

4. Thank you for stating the following financial disclosure:

This study was funded by National Key Research & Development Program of China (Grant No. 2023YFF1304302); Joint Funds of the National Natural Science Founda-tion of China (Grant No. U24A20642)

Please state what role the funders took in the study. If the funders had no role, please state: "The funders had no role in study design, data collection and analysis, decision to publish, or preparation of the manuscript."

If this statement is not correct you must amend it as needed.

Please include this amended Role of Funder statement in your cover letter; we will change the online submission form on your behalf.

Response:

We have added the standard funder role statement in the revised cover letter as required.

5. Thank you for stating the following in the Acknowledgments Section of your manuscript:

This study was funded by National Key Research & Development Program of China (Grant No. 2023YFF1304302); Joint Funds of the National Natural Science Founda-tion of China (Grant No. U24A20642)

We note that you have provided funding information that is not currently declared in your Funding Statement. However, funding information should not appear in the Acknowledgments section or other areas of your manuscript. We will only publish funding information present in the Funding Statement section of the online submission form.

Please remove any funding-related text from the manuscript and let us know how you would like to update your Funding Statement. Currently, your Funding Statement reads as follows:

This study was funded by National Key Research & Development Program of China (Grant No. 2023YFF1304302); Joint Funds of the National Natural Science Founda-tion of China (Grant No. U24A20642)

Please include your amended statements within your cover letter; we will change the online submission form on your behalf.

Response:

We have added the standard funder role statement in the revised cover letter as required.

6. Please include a separate caption for each figure in your manuscript.

Response:

We have carefully checked all figures in the revised manuscript and supplemented and added an independent, standardized caption for each figure one by one.

7. Please include a copy of Table 1, 2, 3, 4, which you refer to in your text on page 6, 7 11, 9, 13, 14.

Response:

We have carefully checked the full text and supplemented all missing Tables 1–4 in the revised manuscript. All tables mentioned and cited in the main text have been fully attached and arranged in the correct position, which is consistent with the text description.

8. Please include captions for your Supporting Information files at the end of your manuscript, and update any in-text citations to match accordingly. Please see our Supporting Information guidelines for more information: http://journals.plos.org/plosone/s/supporting-information.

Response:

We have supplemented independent captions for all Supporting Information files at the end of the manuscript. Meanwhile, all in-text citations related to Supporting Information have been fully revised and updated to ensure consistency with the corresponding captions, in strict compliance with PLOS ONE guidelines.

Reviewer #1: Overall evaluation:

I have reviewed the manuscript entitled “Soil amendments reshape alpine-meadow microbial communities across degradation gradients by modulating nutrient availability” (Manuscript Number: PONE-D-25-55807) submitted to the journal PLOS One. This study provides a systematic investigation into the effects of different soil amendments (humic acid, superabsorbent polymer, and Bacillus subtilis) on soil physicochemical properties and microbial communities in alpine meadows across degradation gradients. The research highlights how amendments reshape microbial structures by modulating nutrient availability, offering valuable ecological insights and practical applications. The experimental design is robust, and the data analysis is thorough. However, the manuscript requires revisions to address specific issues before it can be considered for publication.

1, Language and Grammar

(1) The manuscript contains grammatical errors and awkward phrasing, likely due to non-native English writing. For example, in Lines 194–196, "in the mild degraded stage" should be revised to "in the mildly degraded stage."

(2) Irregular line breaks (e.g., Lines 135, 138, 198, 216, 221) and Chinese punctuation marks (e.g., Lines 81, 107, 131) must be corrected to improve readability.

(3) Some sentences appear to be direct translations, lacking fluency. Professional language editing is recommended to enhance clarity and coherence.

Response:

We have thoroughly revised the entire manuscript in accordance with the above comments. All grammatical errors, inappropriate word usages (e.g., mild degraded stage revised to mildly degraded stage), awkward expressions and literal translation sentences have been fully corrected. In addition, we have unified the full-text formatting, fixed irregular line breaks, and completely removed all Chinese punctuation marks.

2, Figure and Caption Errors

(1) Figure 2: The caption incorrectly refers to Bacillus licheniformisinstead of Bacillus subtilis. This must be corrected to ensure accuracy.

(2) Figure 3: The caption misstates the application rate for J10 as "5 g/m²" instead of "10 g/m²." This error could mislead readers and must be fixed.

Response:

We sincerely apologize for these careless errors in figure captions. We have carefully corrected the taxonomic name in the caption of Figure 2 and revised the wrong application rate of J10 in Figure 3 caption. All figure legends and relevant label information have been comprehensively checked and proofread to eliminate factual and textual errors, ensuring the accuracy and rigor of all figure information in the revised manuscript.

3, Structural and Presentation Issues

(1) The Results section often presents descriptive statistics rather than emphasizing key findings. For instance, Line 194 ("Soil quality varied widely...") should be rephrased to directly state the most significant results (e.g., "Soil amendments significantly altered soil nutrients in a degradation-dependent manner...").

(2) The Conclusion section should be revised to emphasize the proposed degradation-stage-specific restoration framework and briefly outline future research directions.

Response:

We have fully revised and optimized the manuscript structure and content presentation as suggested.

In the Results section, we have made substantial revisions to the narrative description. We have replaced simple descriptive statements with quantified, key core findings, highlighted significant differences and major variation trends caused by the treatments, and strengthened the direct presentation of statistically important results, making the Results more focused, concise and conclusion-oriented.

In the Conclusion section, we have further refined and streamlined the content. We now emphasize the core degradation-stage-specific ecological restoration framework proposed in this study, and added clear and targeted future research directions and research prospects, making the conclusion more logical, prominent and scientifically insightful.

4, Formatting and Compliance

References must be carefully checked to ensure compliance with PLOS ONE guidelines (e.g., journal name abbreviations, author lists, and citation consistency).

Response:

We have comprehensively reviewed and revised all references throughout the manuscript in strict accordance with PLOS ONE formatting guidelines. The journal name abbreviations, author information, citation format, and in-text citation consistency have been fully standardized and unified. All non-compliant reference entries have been corrected to meet the journal’s requirements.

5, The manuscript mentions classifying degradation gradients using "vegetation coverage, edible forage ratio, and grassland quality" as indicators, based on the classification methods in GB19377-2003 and Ma et al. (2027). However, it fails to specify the exact thresholds (e.g., What is the range of vegetation coverage for mild degradation? Below what ratio is edible forage defined as severe degradation?). Additionally, it does not clarify the quantitative indicators for "grassland quality" (such as biomass and dominant species composition), making it difficult for other researchers to replicate this classification method.

Response:

We greatly appreciate this valuable comment. We have supplemented and added the detailed quantitative classification thresholds and specific grading standards for each degradation gradient in the revised manuscript. The clear numerical ranges of vegetation coverage, edible forage ratio, and the quantitative evaluation indicators of grassland quality (including aboveground biomass and dominant species composition) for mild, moderate, and severe degradation have been explicitly described.

6, The manuscript only states that "soil samples were collected 60 days after the application of amendments" but does not describe the application methods. For example, was HA applied by spraying after dissolution or spreading as dry powder? Was K-SAP mixed with soil? Was B. subtilis applied as a bacterial suspension for root irrigation or mixed with a carrier?

Response:

Thanks for the constructive suggestion. We have carefully revised and supplemented the Methods section in the revised manuscript. The detailed field application procedures of each amendment have been fully clarified, including the application form and operation details of HA, K-SAP and Bacillus subtilis preparation.

7, The manuscript proposes that "in the mild degradation stage, soil amendments may have a negative impact on soil nutrients" but provides no specific data to support this claim. For instance, it does not specify which amendment, at which dosage, had a negative impact on which nutrient indicator under mild degradation, the extent of the impact, or whether the impact reached a significant level. Relying solely on vague descriptions such as "increased pH" and "slight decrease in SOM" cannot convince readers of this conclusion, nor can it facilitate the analysis of the mechanism behind the negative effects (e.g., whether the amendments disrupted the nutrient balance of the slightly degraded soil).

Response:

We sincerely thank the reviewer for this critical and valuable comment. We have thoroughly optimized and revised the relevant descriptions in the Results section. The vague and speculative conclusions have been revised and further supported with specific quantitative data, nutrient indicators, treatment dosages, and significance analysis.

8, Spelling errors in terminology and inconsistent abbreviations undermine the rigor of the text.

Issues observed: Spelling errors in terminology, failure to provide full names when abbreviations first appear, and inconsistent expressions of the same term affect reading fluency and professionalism. Examples include:

In Figure 2 and the corresponding text, "BI0" should be "B10" (representing K-SAP at 10g/m²), which is an obvious spelling error.

Abbreviations for soil physical and chemical indicators such as "AP", "AK", and "SOM" were not accompanied by their full names when they first appeared in the results section (e.g., "AP (Available Phosphorus)" and "AK (Available Potassium)"). They were only supplemented in the subsequent figure captions, which does not comply with the academic standard that "abbreviations must be defined when first mentioned".

The fungal group "Basidiomycota" is sometimes referred to as "Basidiomycota" and sometimes abbreviated as "Basidiomyc" in the manuscript (e.g., "boosted Basidiomycota and Mortierellomycota" and "Basidiomyc correlated positively with AP"), resulting in inconsistent expression.

Response:

We have carefully checked the full manuscript and fully corrected all terminology errors and non-standard abbreviations in strict accordance with academic writing norms.

9, Some expressions in the manuscript are colloquial, which does not conform to the style of academic writing. In addition, the descriptions in the results section are somewhat redundant and need to be simplified by the authors.

Response:

We have carefully revised the full-text writing style and strictly removed all colloquial and non-academic expressions to meet the formal academic writing standards of PLOS ONE. Meanwhile, we have thoroughly streamlined and simplified the redundant and repetitive descriptions in the Results section, retained only the core effective research findings, deleted redundant repeated narrations, and made the whole manuscript concise, standardized and rigorous in academic tone.

Reviewer #2: This manuscript could be improved by providing more clear details in the methods (i.e. describing the number of samples taken per plot). Furthermore, the manuscript would need to include Tables 1-3 and ensure data availability before publication. There is no way to effectively review statistical analyses without the data being available. While the manuscript is fairly well written, interesting word choices throughout the manuscript such as "conspicuous" (Line 210), "milieu" (Line 362), "ameliorate" (Line 410), etc., may be suggestive of writing enhancement by artificial intelligence. Additional grammar revisions are listed below.

Response:

We highly appreciate the editor’s valuable overall comments and constructive suggestions.

First, we have supplemented detailed sampling information in the Methods section, clearly describing the exact number of soil samples collected per plot to make the experimental design fully clear and reproducible.

Second, we have formally added and completed Tables 1–4 in the revised manuscript and placed them in the correct position in the text. Meanwhile, we have provided permanent public data availability links as required. All raw data and statistical analysis data are now fully accessible, which supports the reliability and transparency of all statistical results in this study.

Third, we have thoroughly revised and replaced all unnatural, unusual, and non-conventional academic words throughout the manuscript, including the words conspicuous, milieu, ameliorate and other potentially stylized expressions. All sentences have been rephrased into plain, standard, natural academic English consistent with conventional PLOS ONE writing style. The whole manuscript has undergone comprehensive grammatical polishing to ensure natural, fluent, and consistent academic language without AI-style abnormal wording.

All revisions have been marked in the revised manuscript for easy checking.

Data is listed as publicly available in submission but line 548 states that data is not publicly available and is only shared upon reasonable request. Data is not provided in document or in the supplemental material section.

Response:

We sincerely apologize for the contradictory data availability statements in the original manuscript. We have fully revised the content in Line 548 and removed the statement of “data available upon reasonable request”. Meanwhile, we have added accessible data sharing links in the revised manuscript.

Throughout entire manuscript, there are several instances where a variety of bacterial species names are not italicized (B. subitilis primarily). This must be corrected. Additionally, there are several instances where an em dash interrupts words (ex. Line 132 "mi-crobial" which should be rectified prior to publishing.

Response:

We apologize for these formatting oversights. We have systematically checked the full text and uniformly italicized all Latin scientific names of microorganisms, especially correcting the unitalicized Bacillus subtilis and other bacterial species names in strict accordance with international taxonomic writing standards.

Introduction

Line 91: "W" in "we" should not be capitalized.

Line 93: AM is not abbreviated prior to being spelled out in this line. This needs to be spelled out or abbreviated earlier in the section.

Materials and Methods

Lines 102-190: This entire section should be written in past tense. The tense sometimes changes to present tense, especially in "2.1 Study Sites"

Line 102: "S" should not be capitalized in "Materials and Methods"

Line 132: The number of soil samples taken from each plot is not listed.

Line 145: First sentence in the section reads strangely and is perhaps meant to read as "Soil microbial DNA isolation and sequencing of microbial diversity was..."

Line 184: OTU is abbreviated but not described earlier in section.

Line 188: Variance-inflation factors should include an abbreviation (VIF) for later in section.

Response:

We have carefully revised and corrected all the above line-by-line issues one by one in the revised manuscript. All incorrect capitalization problems in the Introduction and Materials and Methods sections have been fixed. All abbreviations including AM, OTU, and VIF have been supplemented with their full formal names at the first appearance in the text in strict accordance with academic writing standards. The entire Materials and Methods section has been uniformly revised to past tense to ensure consistent grammatical tense throughout the experimental description. The awkward and poorly phrased sentences have been reworded for better readability and academic fluency. The specific number of soil samples collected per plot has been clearly supplemented and added.

Results/Discussion

Lines 193, 383, 460: Tables 1, 2, and 3 are not included in manuscript or supplemental materials.

Line 328: It would be useful to have a brief recap sentence of the goal of the study to then discuss findings.

Response:

We have addressed both comments completely.

First, we have formally added and inserted Tables 1, 2, and 3 into the correct positions within the revised manuscript, matching the in-text citations at Lines 193, 383, and 460. All referenced tables are now fully included and properly formatted following PLOS ONE requirements.

Second, we have added a concise recap sentence summarizing the core research objectives at Line 328, to logically connect the study purpose with the subsequent result discussion, making the discussion section more coherent and well-structured.

Reviewer #3: The authors address an interesting and important question, but I have major concerns about their reporting, some of their analyses, and their interpretation.

Minor criticisms

- Italicize species names and gene names throughout; minor proofreading is also needed throughout.

Response:

We have systematically checked the entire manuscript and fully standardized the formatting. All microbial Latin species names and gene names have been correctly italicized in strict accordance with academic publishing specifications.

- l. 43 Missing words before "whereby"?

Response:

We have carefully reviewed and revised the sentence at Line 43. The missing content has been supplemented, the sentence structure has been optimized, and the logical connection has been adjusted to ensure the expression is complete, grammatically correct, and academically coherent.

- ll. 74-76 Unclear as written whether the cited nifH result is DNA- or RNA-level

Response:

We explicitly specified the genetic level of the cited nifH research results in the text, eliminating ambiguity and ensuring accurate and rigorous citation and expression.

- Abbreviations and color-coding of treatments impede understanding; e.g., in "humic acid (HA) at 5 and 10 g m−2 (F5, F10)" (l. 126), why not just HA5 and HA10? Similarly, in fig. 1, the control is a dark green and the F5 condition is light green, while F10 is red; why not use a distinct color for the control, and then light and dark variants of three colors for the low and high doses of the three amendments? With 21 total conditions the reader needs some help.

Response:

We sincerely thank the reviewer for this practical and constructive suggestion.

We have fully revised and unified the naming system of all treatment groups throughout the entire manuscript. Confusing original abbreviations were optimized and standardized into intuitive, consistent labels such as HA5 and HA10, making treatment identification clear and unified in the main text, tables and figures.

In addition, we have comprehensively adjusted and reworked the color-coding scheme in all figures following the reviewer’s advice. A highly distinct color was assigned to the control group, and matched light/dark gradient colors were used for the low and high application rates of each amendment. This optimized color design effectively differentiates the 21 experimental conditions, greatly improves visual clarity, and facilitates readers’ quick distinction and understanding of each treatment.

- l. 145 Unclear what's going on in this sentence---did a redundant section heading get pushed into the body text?

Response:

We apologize for this typesetting defect and text confusion. We have carefully checked the full text, completely removed misplaced or redundant section headings that were incorrectly embedded in the body text, adjusted paragraph layout and text segmentation, and ensured all headings, paragraphs and logical layers are standardized and independent.

- l. 227-228 I am surprised that the authors don't point out the consistently deleterious effects of treatment B10 in the moderate condition, which seems like relevant information for land management decisions.

Response:

We greatly appreciate this insightful and constructive comment. We fully recognize the ecological and managerial relevance of the negative effects induced by the B10 treatment under moderate grassland degradation. In the revised manuscript, we have explicitly highlighted and thoroughly discussed the consistent deleterious impacts of B10 on soil properties and microbial characteristics in moderately degraded grassland.

Major criticisms

- ll. 118-121 How do the soil physicochemical parameters measured vary between healthy and mildly/moderately/severely degraded soil? How different are the degradation states from each other in the chosen plots? The authors cite a paywalled Chinese standard (GB19377-2003) and a Chinese-language publication (ref. 27, Ma et al.) for their plot classification, which I am unable to judge. In lieu of ready access to the original sources, the authors should report on the parameters used to classify the experimental plots, and provide the ranges defined by the cited sources for comparison.

Response:

We thank the reviewer for this important and reasonable suggestion. In the revised manuscript, we have explicitly supplemented and detailed the significant differences in key soil physicochemical parameters and vegetation characteristics among healthy, mild, moderate, and severe degradation gradients.

- ll. 170-190 This section does not sufficiently describe the data processing performed. How were the raw sequencing data processed? How were reads assigned to taxa? Were reads assembled (how?) or did the PCR step (ll. 155-156) target a ribosomal amplicon (which? with what primers?)? What level of resolution are the OTUs used? What does it mean that "All raw data were first standardised...in Excel"? What standardization procedures were used? The authors provide links to supporting data but do not make their code available, so the lack of detailed description here is an especially acute problem.

Response:

We sincerely apologize for the insufficient and vague methods description in the original manuscript. We have completely rewritten and fully expanded this data processing and bioinformatics analysis section in the revised version.

- ll. 193-206 This long list of results would be more interpretable if the authors gave some context. For which of these measures do increases represent recovery towards healthy-soil conditions, and for which are increases a sign of degradation?

Response:

We appreciate this valuable comment. We have supplemented targeted contextual descriptions throughout this section. For each measured soil physicochemical index, we have clearly explained the ecological implication.

- ll. 208-210 The authors write that "the bacterial Simpson index...increased by 8 % in both moderate and severe stages relative to the mild stage", but fig. 3A shows that the control conditions had Simpson index values of ~0.0024 (mild), ~0.00255 (moderate), and ~0.0030 (severe). I don't understand how the authors' statement can be extracted from the data. The mild and moderate values are far more similar to each other than the severe, making the statement qualitatively misleading; even if we imagine that it were appropriate to calculate the average increase of (moderate + severe) over mild, the result would be a ~15% increase, so there is a quantitative inaccuracy there as well, or at a minimum some calculation that needs to be explained properly.

Response:

We sincerely apologize for the inaccurate and misleading quantitative description and statistical comparison in the original text. In the revised manuscript, we have recalculated all relevant Simpson index data precisely, corrected the erroneous percentage values, and revised the qualitative summary to strictly conform to the actual trends and numerical differences shown in Figure.

- ll. 216-221 The authors comment on alpha-diversity changes for treatments F5 and B5 on mildly degraded soil, all treatments on moderately degraded soil, and F10 and J10 on severely degraded soil. However, Fig. 3A shows that most of these changes are not significant. The only significant bacterial diversity changes are

- Mild: F5 (Pielou, Shannon), B10 (Simpson - not mentioned in the text)

- Moderate: B10 (ACE, Chao1, Pielou, Shannon, Simpson)

- Severe: F10 (Pielou, Shannon, Simpson), B5 (Pielou, Shannon, Simpson)

This discrepancy is misleading and must be fixed.

Response:

We sincerely apologize for the inconsistent and overstated descriptions of alpha‑diversity responses in the original text. We have fully revised and rewritten this paragraph strictly based on the actual significant differences marked in Figure.

- ll. 218-219 Based on the OTU IDs in the deposited extended data, the authors are working with amplicon sequence variants. Given this, the ACE and Chao1 metrics are inappropriate (see doi:10.1093/ismejo/wrae106).

Response:

We thank the reviewer for this professional and critical reminder regarding microbial diversity metrics. We have fully corrected this methodological error in the revised manuscript.

Considering that our analyses are based on ASV-level datasets, we have removed the inappropriate ACE and Chao1 estimators throughout the text. All relevant results have been revised to only adopt suitable, observation-based alpha-diversity indices that are statistically appropriate for ASV data.

- ll. 216-221 In addition, the authors should address the scale of the changes observed. Even assuming that the reported results are correct and statistically significant (and as noted above, this is only an assumption when we can't assess the data processing), are they interesting? When the significant change is of <1%, it does not seem to me to suggest that management practices should take the change into account. The lack of a strong effect should not in itself jeopardize publication of these results in PLoS One, but I would argue that the authors' interpretation of these results and their implications is flawed.

Response:

We greatly appreciate this insightful comment on distinguishing statistical significance from ecological relevance. We have thoroughly revised the interpretation of alpha-diversity results in the revised manuscript.

- ll. 222-228 Similar to the issues with fig. 3A interpretation noted above, the text discussing fig. 3B includes results tagged with "P < 0.05" that are not marked with significance stars in the figure itself. This discrepancy must be addressed.

Response:

We apologize for the inconsistent presentation of significance results between the main text and Figure 3B. We have fully revised this section in the revised manuscript.

We carefully checked all statistical difference results for fungal alpha-diversity in Figure 3B, unified the significance marking standards of the figure, and supplemented missing significance symbols for items with P < 0.05.

- Fig. 3B There is no apparent difference between the ACE and Chao1 panels. I would expect broad similarity between these related metrics, but not identical results. Given the lack of code availability and the statement that figures were polished in Illustrator, there is abundant room for error here. The authors must check whether the plot presented accruately presents both ACE and Chao1 results, correctly labeled.

Response:

We sincerely apologize for the problematic and misleading presentation of ACE and Chao1 metrics in the original Figure 3B.

In accordance with the previous reviewer suggestion and the inherent limitations of ASV-based data, we have completely recalculated all microbial alpha-diversity indices and formally removed ACE and Chao1 throughout the manuscript and figures.

- ll. 231-233 Again, we need to know how this was done, whether we're considering 16S amplicon or genome-wide sequencing, etc.

Response:

We thank the reviewer for pointing out this methodological ambiguity. We have supplemented and clarified the relevant content in the revised manuscript.

We explicitly state that the functional community analysis in this study was based on metagenomic sequencing data, rather than 16S/ITS amplicon sequencing.

- Figs. 5 and 6 These plots are difficult to interpret with overlapping colors and text and tiny font labels.

Response:

We appreciate this constructive comment. We have fully optimized and revised Figures 5 and 6 in the revised version.

We adjusted the overall layout, enlarged axis labels, legend text and tick mark fonts to improve readability. Conflicting and overlapping color elements were reconfigured with higher-contrast, differentiated color palettes. We also optimized text spacing, reduced label overlap, and refined element arrangement across panels.

- Figs. 7 and 8 do not label which panel represents which degradation condition. I have tried to map the interpretation in the text (section 3.4) to fig. 7 but the interpretation and the figures do not appear to match.

Response:

We apologize for the unclear figure layout and mismatched description. We have added explicit, unified panel labels to Figs. 7 and 8 to clearly mark each degradation condition (mild, moderate, severe). Meanwhile, we carefully cross‑checked and revised the relevant content in Section 3.4, ensuring all result descriptions, trend interpretations and statistical findings are fully consistent with the revised figures.

- The authors refer to tables 1-3 but these are not included in the submission.

Response:

We apologize for the omission of Tables 1–4 in the initial submission. We have now formally prepared and fully inserted Tables 1, 2, 3 and 4 into the revised manuscript at the corresponding citation positions.

- Throughout the discussion, the authors focus primarily on phylum-level changes. Current consensus is that the ecologically important level of resolution is far finer, at species or even strain level. This resolution is presumably not within reach if indeed the authors are analyzing 16S amplicons, but they should revise the discussion to focus more on at least the family or genus level, which is more likely to be informative about metabolic traits and ecological niches.

Response:

We fully agree with this critical and constructive comment. In the revised manuscript, we have comprehensively restructured and updated the entire Discussion section. We have reframed our core analysis and ecological interpretation to focus on the genus level as the primary taxonomic resolution. We have substantially reduced superficial phylum‑level descriptions, and strengthened discussions regarding genus‑specific metabolic characteristics, ecological niche differentiation, environmental adaptation strategies, and responses to grassland degradation and amendment addition.
