# Supplementary material for: Soil amendments reshape alpine meadow microbial communities across degradation gradients by modulating nutrient availability
Source: PLoS One. 2026 Nov 3;21(11):e0359576. doi: 10.1371/journal.pone.0359576 (PMC13730322; doi:10.1371/journal.pone.0359576)
Supplement: Peer review history

# Peer review: Decision Letter 0

**DOI:** 10.1371/journal.pone.0359576.r001

**Authors:** Tiziana Danise

20 Jan 2026

Dear Dr. Wang,

Please submit your revised manuscript by Mar 06 2026 11:59PM. If you will need more time than this to complete your revisions, please reply to this message or contact the journal office at plosone@plos.org. When you're ready to submit your revision, log on to https://www.editorialmanager.com/pone/ and select the 'Submissions Needing Revision' folder to locate your manuscript file.

- A letter that responds to each point raised by the academic editor and reviewer(s). You should upload this letter as a separate file labeled 'Response to Reviewers'.
- A marked-up copy of your manuscript that highlights changes made to the original version. You should upload this as a separate file labeled 'Revised Manuscript with Track Changes'.
- An unmarked version of your revised paper without tracked changes. You should upload this as a separate file labeled 'Manuscript'.

If you would like to make changes to your financial disclosure, please include your updated statement in your cover letter. Guidelines for resubmitting your figure files are available below the reviewer comments at the end of this letter.

If applicable, we recommend that you deposit your laboratory protocols in protocols.io to enhance the reproducibility of your results. Protocols.io assigns your protocol its own identifier (DOI) so that it can be cited independently in the future. For instructions see: https://journals.plos.org/plosone/s/submission-guidelines#loc-laboratory-protocols. Additionally, PLOS ONE offers an option for publishing peer-reviewed Lab Protocol articles, which describe protocols hosted on protocols.io. Read more information on sharing protocols at https://plos.org/protocols?utm_medium=editorial-email&utm_source=authorletters&utm_campaign=protocols.

We look forward to receiving your revised manuscript.

Kind regards,

Tiziana Danise, PhD

Academic Editor

PLOS One

Journal Requirements:

When submitting your revision, we need you to address these additional requirements.

1. Please ensure that your manuscript meets PLOS ONE’s style requirements, including those for file naming. The PLOS ONE style templates can be found at https://journals.plos.org/plosone/s/file?id=wjVg/PLOSOne_formatting_sample_main_body.pdf and https://journals.plos.org/plosone/s/file?id=ba62/PLOSOne_formatting_sample_title_authors_affiliations.pdf

2. In your Methods section, please provide additional information regarding the permits you obtained for the work. Please ensure you have included the full name of the authority that approved the field site access and, if no permits were required, a brief statement explaining why.

3. When completing the data availability statement of the submission form, you indicated that you will make your data available on acceptance. We strongly recommend all authors decide on a data sharing plan before acceptance, as the process can be lengthy and hold up publication timelines. Please note that, though access restrictions are acceptable now, your entire data will need to be made freely accessible if your manuscript is accepted for publication. This policy applies to all data except where public deposition would breach compliance with the protocol approved by your research ethics board. If you are unable to adhere to our open data policy, please kindly revise your statement to explain your reasoning and we will seek the editor’s input on an exemption. Please be assured that, once you have provided your new statement, the assessment of your exemption will not hold up the peer review process.

4. Thank you for stating the following financial disclosure:

This study was funded by National Key Research & Development Program of China (Grant No. 2023YFF1304302); Joint Funds of the National Natural Science Founda-tion of China (Grant No. U24A20642)

Please state what role the funders took in the study. If the funders had no role, please state: "The funders had no role in study design, data collection and analysis, decision to publish, or preparation of the manuscript."

If this statement is not correct you must amend it as needed.

Please include this amended Role of Funder statement in your cover letter; we will change the online submission form on your behalf.

5. Thank you for stating the following in the Acknowledgments Section of your manuscript:

This study was funded by National Key Research & Development Program of China (Grant No. 2023YFF1304302); Joint Funds of the National Natural Science Founda-tion of China (Grant No. U24A20642)

We note that you have provided funding information that is not currently declared in your Funding Statement. However, funding information should not appear in the Acknowledgments section or other areas of your manuscript. We will only publish funding information present in the Funding Statement section of the online submission form.

Please remove any funding-related text from the manuscript and let us know how you would like to update your Funding Statement. Currently, your Funding Statement reads as follows:

This study was funded by National Key Research & Development Program of China (Grant No. 2023YFF1304302); Joint Funds of the National Natural Science Founda-tion of China (Grant No. U24A20642)

Please include your amended statements within your cover letter; we will change the online submission form on your behalf.

6. Please include a separate caption for each figure in your manuscript.

7. Please include a copy of Table 1, 2, 3, 4, which you refer to in your text on page 6, 7 11, 9, 13, 14.

8. Please include captions for your Supporting Information files at the end of your manuscript, and update any in-text citations to match accordingly. Please see our Supporting Information guidelines for more information: http://journals.plos.org/plosone/s/supporting-information.

9. If the reviewer comments include a recommendation to cite specific previously published works, please review and evaluate these publications to determine whether they are relevant and should be cited. There is no requirement to cite these works unless the editor has indicated otherwise.

[Note: HTML markup is below. Please do not edit.]

Reviewers' comments:

Reviewer’s Responses to Questions

**Comments to the Author**

1. Is the manuscript technically sound, and do the data support the conclusions?

Reviewer #1: Yes

Reviewer #2: Partly

Reviewer #3: No

**********

2. Has the statistical analysis been performed appropriately and rigorously?

Reviewer #1: Yes

Reviewer #2: I Don't Know

Reviewer #3: No

**********

3. Have the authors made all data underlying the findings in their manuscript fully available?

The PLOS Data policy

Reviewer #1: Yes

Reviewer #2: No

Reviewer #3: No

**********

4. Is the manuscript presented in an intelligible fashion and written in standard English?

Reviewer #1: Yes

Reviewer #2: Yes

Reviewer #3: Yes

**********

Reviewer #1: Overall evaluation:

I have reviewed the manuscript entitled “Soil amendments reshape alpine-meadow microbial communities across degradation gradients by modulating nutrient availability” (Manuscript Number: PONE-D-25-55807) submitted to the journal PLOS One. This study provides a systematic investigation into the effects of different soil amendments (humic acid, superabsorbent polymer, and Bacillus subtilis) on soil physicochemical properties and microbial communities in alpine meadows across degradation gradients. The research highlights how amendments reshape microbial structures by modulating nutrient availability, offering valuable ecological insights and practical applications. The experimental design is robust, and the data analysis is thorough. However, the manuscript requires revisions to address specific issues before it can be considered for publication.

1, Language and Grammar

(1) The manuscript contains grammatical errors and awkward phrasing, likely due to non-native English writing. For example, in Lines 194–196, "in the mild degraded stage" should be revised to "in the mildly degraded stage."

(2) Irregular line breaks (e.g., Lines 135, 138, 198, 216, 221) and Chinese punctuation marks (e.g., Lines 81, 107, 131) must be corrected to improve readability.

(3) Some sentences appear to be direct translations, lacking fluency. Professional language editing is recommended to enhance clarity and coherence.

2, Figure and Caption Errors

(1) Figure 2: The caption incorrectly refers to Bacillus licheniformisinstead of Bacillus subtilis. This must be corrected to ensure accuracy.

(2) Figure 3: The caption misstates the application rate for J10 as "5 g/m²" instead of "10 g/m²." This error could mislead readers and must be fixed.

3, Structural and Presentation Issues

(1) The Results section often presents descriptive statistics rather than emphasizing key findings. For instance, Line 194 ("Soil quality varied widely...") should be rephrased to directly state the most significant results (e.g., "Soil amendments significantly altered soil nutrients in a degradation-dependent manner...").

(2) The Conclusion section should be revised to emphasize the proposed degradation-stage-specific restoration framework and briefly outline future research directions.

4, Formatting and Compliance

References must be carefully checked to ensure compliance with PLOS ONE guidelines (e.g., journal name abbreviations, author lists, and citation consistency).

5, The manuscript mentions classifying degradation gradients using "vegetation coverage, edible forage ratio, and grassland quality" as indicators, based on the classification methods in GB19377-2003 and Ma et al. (2027). However, it fails to specify the exact thresholds (e.g., What is the range of vegetation coverage for mild degradation? Below what ratio is edible forage defined as severe degradation?). Additionally, it does not clarify the quantitative indicators for "grassland quality" (such as biomass and dominant species composition), making it difficult for other researchers to replicate this classification method.

6, The manuscript only states that "soil samples were collected 60 days after the application of amendments" but does not describe the application methods. For example, was HA applied by spraying after dissolution or spreading as dry powder? Was K-SAP mixed with soil? Was B. subtilis applied as a bacterial suspension for root irrigation or mixed with a carrier?

7, The manuscript proposes that "in the mild degradation stage, soil amendments may have a negative impact on soil nutrients" but provides no specific data to support this claim. For instance, it does not specify which amendment, at which dosage, had a negative impact on which nutrient indicator under mild degradation, the extent of the impact, or whether the impact reached a significant level. Relying solely on vague descriptions such as "increased pH" and "slight decrease in SOM" cannot convince readers of this conclusion, nor can it facilitate the analysis of the mechanism behind the negative effects (e.g., whether the amendments disrupted the nutrient balance of the slightly degraded soil).

8, Spelling errors in terminology and inconsistent abbreviations undermine the rigor of the text.

Issues observed: Spelling errors in terminology, failure to provide full names when abbreviations first appear, and inconsistent expressions of the same term affect reading fluency and professionalism. Examples include:

In Figure 2 and the corresponding text, "BI0" should be "B10" (representing K-SAP at 10g/m²), which is an obvious spelling error.

Abbreviations for soil physical and chemical indicators such as "AP", "AK", and "SOM" were not accompanied by their full names when they first appeared in the results section (e.g., "AP (Available Phosphorus)" and "AK (Available Potassium)"). They were only supplemented in the subsequent figure captions, which does not comply with the academic standard that "abbreviations must be defined when first mentioned".

The fungal group "Basidiomycota" is sometimes referred to as "Basidiomycota" and sometimes abbreviated as "Basidiomyc" in the manuscript (e.g., "boosted Basidiomycota and Mortierellomycota" and "Basidiomyc correlated positively with AP"), resulting in inconsistent expression.

9, Some expressions in the manuscript are colloquial, which does not conform to the style of academic writing. In addition, the descriptions in the results section are somewhat redundant and need to be simplified by the authors.

Reviewer #2: This manuscript could be improved by providing more clear details in the methods (i.e. describing the number of samples taken per plot). Furthermore, the manuscript would need to include Tables 1-3 and ensure data availability before publication. There is no way to effectively review statistical analyses without the data being available. While the manuscript is fairly well written, interesting word choices throughout the manuscript such as "conspicuous" (Line 210), "milieu" (Line 362), "ameliorate" (Line 410), etc., may be suggestive of writing enhancement by artificial intelligence. Additional grammar revisions are listed below.

Data is listed as publicly available in submission but line 548 states that data is not publicly available and is only shared upon reasonable request. Data is not provided in document or in the supplemental material section.

Throughout entire manuscript, there are several instances where a variety of bacterial species names are not italicized (B. subitilis primarily). This must be corrected. Additionally, there are several instances where an em dash interrupts words (ex. Line 132 "mi-crobial" which should be rectified prior to publishing.

Introduction

Line 91: "W" in "we" should not be capitalized.

Line 93: AM is not abbreviated prior to being spelled out in this line. This needs to be spelled out or abbreviated earlier in the section.

Materials and Methods

Lines 102-190: This entire section should be written in past tense. The tense sometimes changes to present tense, especially in "2.1 Study Sites"

Line 102: "S" should not be capitalized in "Materials and Methods"

Line 132: The number of soil samples taken from each plot is not listed.

Line 145: First sentence in the section reads strangely and is perhaps meant to read as "Soil microbial DNA isolation and sequencing of microbial diversity was..."

Line 184: OTU is abbreviated but not described earlier in section.

Line 188: Variance-inflation factors should include an abbreviation (VIF) for later in section.

Results/Discussion

Lines 193, 383, 460: Tables 1, 2, and 3 are not included in manuscript or supplemental materials.

Line 328: It would be useful to have a brief recap sentence of the goal of the study to then discuss findings.

This reviewer recommends major revision prior to publishing. A useful review of the manuscript is impossible without the data being available and Tables missing from throughout the manuscript.

Reviewer #3: The authors address an interesting and important question, but I have major concerns about their reporting, some of their analyses, and their interpretation.

Minor criticisms

- Italicize species names and gene names throughout; minor proofreading is also needed throughout.

- l. 43 Missing words before "whereby"?

- ll. 74-76 Unclear as written whether the cited nifH result is DNA- or RNA-level

- Abbreviations and color-coding of treatments impede understanding; e.g., in "humic acid (HA) at 5 and 10 g m−2 (F5, F10)" (l. 126), why not just HA5 and HA10? Similarly, in fig. 1, the control is a dark green and the F5 condition is light green, while F10 is red; why not use a distinct color for the control, and then light and dark variants of three colors for the low and high doses of the three amendments? With 21 total conditions the reader needs some help.

- l. 145 Unclear what’s going on in this sentence---did a redundant section heading get pushed into the body text?

- l. 227-228 I am surprised that the authors don't point out the consistently deleterious effects of treatment B10 in the moderate condition, which seems like relevant information for land management decisions.

Major criticisms

- ll. 118-121 How do the soil physicochemical parameters measured vary between healthy and mildly/moderately/severely degraded soil? How different are the degradation states from each other in the chosen plots? The authors cite a paywalled Chinese standard (GB19377-2003) and a Chinese-language publication (ref. 27, Ma et al.) for their plot classification, which I am unable to judge. In lieu of ready access to the original sources, the authors should report on the parameters used to classify the experimental plots, and provide the ranges defined by the cited sources for comparison.

- ll. 170-190 This section does not sufficiently describe the data processing performed. How were the raw sequencing data processed? How were reads assigned to taxa? Were reads assembled (how?) or did the PCR step (ll. 155-156) target a ribosomal amplicon (which? with what primers?)? What level of resolution are the OTUs used? What does it mean that "All raw data were first standardised...in Excel"? What standardization procedures were used? The authors provide links to supporting data but do not make their code available, so the lack of detailed description here is an especially acute problem.

- ll. 193-206 This long list of results would be more interpretable if the authors gave some context. For which of these measures do increases represent recovery towards healthy-soil conditions, and for which are increases a sign of degradation?

- ll. 208-210 The authors write that "the bacterial Simpson index...increased by 8 % in both moderate and severe stages relative to the mild stage", but fig. 3A shows that the control conditions had Simpson index values of ~0.0024 (mild), ~0.00255 (moderate), and ~0.0030 (severe). I don't understand how the authors' statement can be extracted from the data. The mild and moderate values are far more similar to each other than the severe, making the statement qualitatively misleading; even if we imagine that it were appropriate to calculate the average increase of (moderate + severe) over mild, the result would be a ~15% increase, so there is a quantitative inaccuracy there as well, or at a minimum some calculation that needs to be explained properly.

- ll. 216-221 The authors comment on alpha-diversity changes for treatments F5 and B5 on mildly degraded soil, all treatments on moderately degraded soil, and F10 and J10 on severely degraded soil. However, Fig. 3A shows that most of these changes are not significant. The only significant bacterial diversity changes are

- Mild: F5 (Pielou, Shannon), B10 (Simpson - not mentioned in the text)

- Moderate: B10 (ACE, Chao1, Pielou, Shannon, Simpson)

- Severe: F10 (Pielou, Shannon, Simpson), B5 (Pielou, Shannon, Simpson)

This discrepancy is misleading and must be fixed.

- ll. 218-219 Based on the OTU IDs in the deposited extended data, the authors are working with amplicon sequence variants. Given this, the ACE and Chao1 metrics are inappropriate (see doi:10.1093/ismejo/wrae106).

- ll. 216-221 In addition, the authors should address the scale of the changes observed. Even assuming that the reported results are correct and statistically significant (and as noted above, this is only an assumption when we can't assess the data processing), are they interesting? When the significant change is of <1%, it does not seem to me to suggest that management practices should take the change into account. The lack of a strong effect should not in itself jeopardize publication of these results in PLoS One, but I would argue that the authors' interpretation of these results and their implications is flawed.

- ll. 222-228 Similar to the issues with fig. 3A interpretation noted above, the text discussing fig. 3B includes results tagged with "P < 0.05" that are not marked with significance stars in the figure itself. This discrepancy must be addressed.

- Fig. 3B There is no apparent difference between the ACE and Chao1 panels. I would expect broad similarity between these related metrics, but not identical results. Given the lack of code availability and the statement that figures were polished in Illustrator, there is abundant room for error here. The authors must check whether the plot presented accruately presents both ACE and Chao1 results, correctly labeled.

- ll. 231-233 Again, we need to know how this was done, whether we're considering 16S amplicon or genome-wide sequencing, etc.

- Figs. 5 and 6 These plots are difficult to interpret with overlapping colors and text and tiny font labels.

- Figs. 7 and 8 do not label which panel represents which degradation condition. I have tried to map the interpretation in the text (section 3.4) to fig. 7 but the interpretation and the figures do not appear to match.

- The authors refer to tables 1-3 but these are not included in the submission.

- Throughout the discussion, the authors focus primarily on phylum-level changes. Current consensus is that the ecologically important level of resolution is far finer, at species or even strain level. This resolution is presumably not within reach if indeed the authors are analyzing 16S amplicons, but they should revise the discussion to focus more on at least the family or genus level, which is more likely to be informative about metabolic traits and ecological niches.

**********

what does this mean?). If published, this will include your full peer review and any attached files.

If you choose “no”, your identity will remain anonymous but your review may still be made public.

**Do you want your identity to be public for this peer review?**  For information about this choice, including consent withdrawal, please see our Privacy Policy

Reviewer #1: No

Reviewer #2: No

Reviewer #3: No

**********

[NOTE: If reviewer comments were submitted as an attachment file, they will be attached to this email and accessible via the submission site. Please log into your account, locate the manuscript record, and check for the action link "View Attachments". If this link does not appear, there are no attachment files.]

To ensure your figures meet our technical requirements, please review our figure guidelines: https://journals.plos.org/plosone/s/figures

You may also use PLOS’s free figure tool, NAAS, to help you prepare publication quality figures: https://journals.plos.org/plosone/s/figures#loc-tools-for-figure-preparation.

NAAS will assess whether your figures meet our technical requirements by comparing each figure against our figure specifications.

# Author comment: Author response to Decision Letter 1

**DOI:** 10.1371/journal.pone.0359576.r002

29 Apr 2026

Dear Editor and Reviewers,

We thank the editor and reviewers for their constructive comments and suggestions, which have greatly helped improve the quality of our manuscript. We have carefully addressed all comments point-by-point. A detailed response letter is provided as a supplementary file, and all revisions have been highlighted in the revised manuscript.

Sincerely,

The authors

Attachment Submitted filename: Response to Reviewers.docx

# Peer review: Decision Letter 1

**DOI:** 10.1371/journal.pone.0359576.r003

**Authors:** Massimiliano Cardinale

8 Jul 2026

Dear Dr. Wang,

Thank you for submitting your manuscript to PLOS ONE. After careful consideration, we feel that it has merit but does not fully meet PLOS ONE’s publication criteria as it currently stands. Therefore, we invite you to submit a revised version of the manuscript that addresses the points raised during the review process.Please submit your revised manuscript by Aug 22 2026 11:59PM. If you will need more time than this to complete your revisions, please reply to this message or contact the journal office at plosone@plos.org. When you're ready to submit your revision, log on to https://www.editorialmanager.com/pone/ and select the 'Submissions Needing Revision' folder to locate your manuscript file.

- A letter that responds to each point raised by the academic editor and reviewer(s). You should upload this letter as a separate file labeled 'Response to Reviewers'.
- A marked-up copy of your manuscript that highlights changes made to the original version. You should upload this as a separate file labeled 'Revised Manuscript with Track Changes'.
- An unmarked version of your revised paper without tracked changes. You should upload this as a separate file labeled 'Manuscript'.

If you would like to make changes to your financial disclosure, please include your updated statement in your cover letter. Guidelines for resubmitting your figure files are available below the reviewer comments at the end of this letter.

If applicable, we recommend that you deposit your laboratory protocols in protocols.io to enhance the reproducibility of your results. Protocols.io assigns your protocol its own identifier (DOI) so that it can be cited independently in the future. For instructions see: https://journals.plos.org/plosone/s/submission-guidelines#loc-laboratory-protocols. Additionally, PLOS ONE offers an option for publishing peer-reviewed Lab Protocol articles, which describe protocols hosted on protocols.io. Read more information on sharing protocols at https://plos.org/protocols?utm_medium=editorial-email&utm_source=authorletters&utm_campaign=protocols.

As the corresponding author, your ORCID iD is verified in the submission system and will appear in the published article. PLOS supports the use of ORCID, and we encourage all coauthors to register for an ORCID iD and use it as well. Please encourage your coauthors to verify their ORCID iD within the submission system before final acceptance, as unverified ORCID iDs will not appear in the published article. *Only*  the individual author can complete the verification step; PLOS staff *cannot*  verify ORCID iDs on behalf of authors.

We look forward to receiving your revised manuscript.

Kind regards,

Massimiliano Cardinale, PhD

Academic Editor

PLOS One

Journal Requirements:

If the reviewer comments include a recommendation to cite specific previously published works, please review and evaluate these publications to determine whether they are relevant and should be cited. There is no requirement to cite these works unless the editor has indicated otherwise.

[Note: HTML markup is below. Please do not edit.]

Reviewers' comments:

Reviewer’s Responses to Questions

**Comments to the Author**

Reviewer #3: (No Response)

**********

2. Is the manuscript technically sound, and do the data support the conclusions?

Reviewer #3: Partly

**********

3. Has the statistical analysis been performed appropriately and rigorously?

Reviewer #3: I Don't Know

**********

4. Have the authors made all data underlying the findings in their manuscript fully available?

The PLOS Data policy

Reviewer #3: Yes

**********

5. Is the manuscript presented in an intelligible fashion and written in standard English?

Reviewer #3: No

**********

Reviewer #3: * Minor criticisms

** Previously flagged, as yet uncorrected

- Species name still un-italicized in l. 33, 42, 80, etc.

- Words within a line of text are broken by hyphens in line 131 and elsewhere throughout

- Many minor typos around punctuation, e.g. at ll. 201-206; many nonstandard punctuation characters throughout

- Fig. 1 abbreviations changed as suggested but color-coding is unchanged, in conflict with what the authors state in their response letter

** New

- ll. 151-153: New information about amendment application is helpful but should be in the past tense (describing what was done) rather than the imperative case (telling the reader what to do).

- Table 3 reports observed species count to the hundredths place. The last two significant digits reported are not meaningful.

- ll. 579-581: The authors write "In contrast, fungal hyphal networks possess advantages in resource acquisition and niche modification, driving the expansion of fungal communities." It’s not clear to me on what basis they argue that fungal communities are expanding after treatment of mildly degraded soils; the differences in fungal richness reported in fig. 3B are not significant. I'm also not sure in what sense the authors mean "expanding": are they referring to total fungal abundance, or to richness, or some other measure?

- ll. 689-690: The authors conclude section 4.2 with the strong claim that "for severely degraded soils, synergistic use of K-SAP treatment and microbial amendments is required." While I see that these treatments taken individually evoked distinct responses across the microbial groups present, this claim is completely unjustified because the authors did not in fact *test* the combined treatment they say is required.

* Major criticisms

** Previously flagged, as yet uncorrected

- Section 3.2 of results continues to highlight results that are not statistically significant. In some cases this is flagged, though not in all (e.g. for fungi, the effects of K10 treatment in moderately degraded soils).

- The LEfSe trees (figs. 5, 6) have been revised somewhat but remain quite difficult to read.

- The data in table 4 do not appear to be consistent with the statement (ll. 479-481) that "overall, as the degree of degradation increased, the explanatory power of environmental factors on the bacterial community gradually strengthened".

- ll. 581-588: although the presentation of alpha diversity data in the revised results section has become more careful about distinguishing between significant and non-significant results, the authors continue to rest their interpretation on non-significant results, as they do in this passage: e.g., "During the moderately degraded stage, all amendment treatments reduced bacterial diversity, with the inhibitory effect of the K10 treatment being significant", followed by a sentence of speculation about the purported pattern seen across treatments. Similarly for fungi in the next sentence: one treatment of moderately degraded soil gave a significant result by one measure, but the authors speculate as though the results were significant across the board.

- ll. 745-747: Further to the previous point, the authors argue that the differing effects of different amendments "impl[y] that targeted regulation of key variables (for example, boosting SOM with B10 or fine-tuning AK and pH with HA5) can thus re-assemble the microbial network." While this *could* be true, and would be terrific to establish firmly, the work presented is certainly not sufficient to support this inference, given the complex and often non-linear emergent dynamics of such systems. In particular, judging whether the microbial network has been "re-assembled" demands knowledge of the community baseline prior to disturbance.

** New

- ll. 184-96: New methods details are presented in a series of sentence fragments and raise a number of new questions. First, the authors state clearly in their response letter that their "analyses are based on ASV-level datasets", but their methods do not describe amplification and do appear to describe shotgun metagenomics. (Later in the response letter, however, the authors appear to contradict themselves by saying "We explicitly state that the functional community analysis in this study was based on metagenomic sequencing data, rather than 16S/ITS amplicon sequencing"---so I am once again confused. If the authors have shotgun metagenomes, I encourage them to make use of them, to do a more detailed genome-centric analysis of the community members that respond to treatment. Such an analysis would be outside the scope of this work, but there is much more to be learned from whole metagenomes than just the taxonomic distribution of the community.) Second, statements such as "selected an appropriate assembly software" are insufficient, and the authors make no mention at all of binning nor of how they defined OTUs. Third, this paragraph is in a section about DNA sequencing but appears (e.g. ll. 188-191, "Aligned the Clean Reads to the contigs using Bowtie2, counted the number of reads mapped to each contig, and then calculated TPM values with R software. Based on the predicted contig/gene relationships, obtained the expression levels of individual genes") to be describing metatranscriptomic mapping and differential expression analysis. If indeed this is about metatranscriptomics, then we need additional details on RNA extraction, rRNA removal (if any), etc., as the extraction and library prep details in the paragraph above just cover DNA.

**********

what does this mean?). If published, this will include your full peer review and any attached files.

If you choose “no”, your identity will remain anonymous but your review may still be made public.

**Do you want your identity to be public for this peer review?**  For information about this choice, including consent withdrawal, please see our Privacy Policy

Reviewer #3: No

**********

[NOTE: If reviewer comments were submitted as an attachment file, they will be attached to this email and accessible via the submission site. Please log into your account, locate the manuscript record, and check for the action link "View Attachments". If this link does not appear, there are no attachment files.]

To ensure your figures meet our technical requirements, please review our figure guidelines: https://journals.plos.org/plosone/s/figures

You may also use PLOS’s free figure tool, NAAS, to help you prepare publication quality figures: https://journals.plos.org/plosone/s/figures#loc-tools-for-figure-preparation.

NAAS will assess whether your figures meet our technical requirements by comparing each figure against our figure specifications.

# Author comment: Author response to Decision Letter 2

**DOI:** 10.1371/journal.pone.0359576.r004

27 Aug 2026

Response to Reviewers

Manuscript ID: PONE-D-25-55807R1

Dear Dr. Cardinale and Reviewer #3,

We sincerely thank you for your careful evaluation of our manuscript and for the constructive comments. We have carefully revised the manuscript to address each point raised. Below, we respond to every comment point by point. Reviewer comments are reproduced in italics, followed by our responses. All revisions in the manuscript are highlighted with tracked changes.

Minor Criticisms

Previously flagged, as yet uncorrected

Comment 1. Species name still un-italicized in l. 33, 42, 80, etc.

Response: We thank the reviewer for catching this. All Latin binomial species names (e.g., Bacillus subtilis, B. subtilis, Kobresia tibetica) and gene symbols throughout the manuscript have been checked and formatted in italics, including the passages previously noted (around ll. 33, 42, and 80) and elsewhere in the text. Phylum-level names, which do not require italicization under the International Code of Nomenclature of Prokaryotes, remain in regular font.

Comment 2. Words within a line of text are broken by hyphens in line 131 and elsewhere throughout.

Response: The manuscript has been professionally language-polished and re-typeset for this revision. All artificial line-break hyphenations (e.g., words split across lines by hard hyphens) have been removed, and the text now flows naturally without such breaks.

Comment 3. Many minor typos around punctuation, e.g. at ll. 201–206; many nonstandard punctuation characters throughout.

Response: We apologize for these errors. The entire manuscript has undergone thorough proofreading and professional English polishing. Punctuation has been standardized throughout, including em dashes, en dashes, commas, semicolons, and special characters (e.g., proper minus signs, multiplication signs, and superscript units). The specific issues around ll. 201–206 and all other nonstandard punctuation characters have been corrected.

Comment 4. Fig. 1 abbreviations changed as suggested but color-coding is unchanged, in conflict with what the authors state in their response letter.

Response: We apologize for this discrepancy in the previous round. In this revision, the color schemes of all figures—including Figure 1—have been genuinely updated. We have adopted a consistent palette with clearly distinguishable colors across treatment groups throughout Figures 1–8. The color assignments are now internally consistent across all figures and match the descriptions in the figure legends.

New

Comment 5. ll. 151–153: New information about amendment application is helpful but should be in the past tense (describing what was done) rather than the imperative case (telling the reader what to do).

Response: We thank the reviewer for this suggestion. The amendment application procedure has been rewritten entirely in the past tense.

Comment 6. Table 3 reports observed species count to the hundredths place. The last two significant digits reported are not meaningful.

Response: We agree. The observed species counts in Table 3 are now reported as integers with standard errors rounded to whole numbers, removing the meaningless decimal places.

Comment 7. ll. 579–581: The authors write "In contrast, fungal hyphal networks possess advantages in resource acquisition and niche modification, driving the expansion of fungal communities." It’s not clear on what basis they argue that fungal communities are expanding after treatment of mildly degraded soils; the differences in fungal richness reported in fig. 3B are not significant. I'm also not sure in what sense the authors mean "expanding": are they referring to total fungal abundance, or to richness, or some other measure?

Response: We thank the reviewer for identifying this unsupported statement. The sentence claiming that fungal communities were "expanding" has been removed. In the revised Discussion (Section 4.2), we now explicitly state that "all changes in fungal α-diversity induced by amendments at the moderate degradation stage were statistically non-significant. Accordingly, no unified directional response pattern of fungal communities to soil amendments could be identified for moderately degraded grassland." For the mild degradation stage, we similarly note that "soil amendments increased fungal diversity and richness, with the K5 treatment presenting the greatest promoting effect, although no significant difference was detected" (Results, Section 3.2). We no longer infer "expansion" (in abundance, richness, or any other measure) from non-significant richness trends.

Comment 8. ll. 689–690: The authors conclude section 4.2 with the strong claim that "for severely degraded soils, synergistic use of K-SAP treatment and microbial amendments is required." While I see that these treatments taken individually evoked distinct responses across the microbial groups present, this claim is completely unjustified because the authors did not in fact test the combined treatment they say is required.

Response: We agree that the original claim overstated what our experiment could support, as no factorial combination of K-SAP and microbial inoculants was directly tested. We have revised this statement accordingly. The prescriptive wording "is required" has been removed.

Major Criticisms

Previously flagged, as yet uncorrected

Comment 9. Section 3.2 of results continues to highlight results that are not statistically significant. In some cases this is flagged, though not in all (e.g. for fungi, the effects of K10 treatment in moderately degraded soils).

Response: We have carefully revised Section 3.2 to ensure that every treatment comparison is explicitly labeled as statistically significant or non-significant.

Comment 10. The LEfSe trees (figs. 5, 6) have been revised somewhat but remain quite difficult to read.

Response: We have substantially redesigned Figures 5 and 6 to improve readability. The circular cladograms have been replaced with horizontal LDA score bar plots, which present the discriminative taxa in a clear, ranked format. To reduce visual clutter, only taxa from phylum to genus level are displayed in the main figures; species-level biomarkers have been moved to Supplementary Figures S2 (bacteria) and S3 (fungi).

Comment 11. The data in table 4 do not appear to be consistent with the statement (ll. 479–481) that "overall, as the degree of degradation increased, the explanatory power of environmental factors on the bacterial community gradually strengthened".

Response: We thank the reviewer for catching this inconsistency. The original statement was imprecise and has been corrected to accurately reflect the data in Table 4.

Comment 12. ll. 581–588: although the presentation of alpha diversity data in the revised results section has become more careful about distinguishing between significant and non-significant results, the authors continue to rest their interpretation on non-significant results, as they do in this passage: e.g., "During the moderately degraded stage, all amendment treatments reduced bacterial diversity, with the inhibitory effect of the K10 treatment being significant", followed by a sentence of speculation about the purported pattern seen across treatments. Similarly for fungi in the next sentence: one treatment of moderately degraded soil gave a significant result by one measure, but the authors speculate as though the results were significant across the board.

Response: We have revised the Discussion (Section 4.2) to eliminate speculation based on non-significant results.

Comment 13. ll. 745–747: Further to the previous point, the authors argue that the differing effects of different amendments "impl[y] that targeted regulation of key variables (for example, boosting SOM with B10 or fine-tuning AK and pH with HA5) can thus re-assemble the microbial network." While this could be true, and would be terrific to establish firmly, the work presented is certainly not sufficient to support this inference, given the complex and often non-linear emergent dynamics of such systems. In particular, judging whether the microbial network has been "re-assembled" demands knowledge of the community baseline prior to disturbance.

Response: We agree that the original claim of "re-assembling the microbial network" was too strong given the data and the absence of pre-disturbance baseline data. We have made the following revisions: (1) The phrase "re-assemble the microbial network" has been changed to "reassemble soil microbial communities," which is a less specific claim more closely aligned with our taxonomic composition data (Section 4.3). (2) The statement is now explicitly framed as an implication rather than a demonstrated conclusion: "This finding implies that targeted regulation of key variables … can reassemble soil microbial communities." (3) We have added a limitations paragraph acknowledging that "this study was based on a single-growing-season field experiment, and we cannot fully capture potential inter-annual variation in soil microbial responses to amendments," and that "future long-term monitoring combined with functional omics is needed to reveal the sustained functional evolution of soil–microbe systems under restoration" (Section 4.3; Conclusions). We agree that definitive demonstration of community (re)assembly would require pre-disturbance baseline data and long-term observation, and we have cited this as an important future direction.

New

Comment 14. ll. 184–96: New methods details are presented in a series of sentence fragments and raise a number of new questions. First, the authors state clearly in their response letter that their "analyses are based on ASV-level datasets", but their methods do not describe amplification and do appear to describe shotgun metagenomics. (Later in the response letter, however, the authors appear to contradict themselves by saying "We explicitly state that the functional community analysis in this study was based on metagenomic sequencing data, rather than 16S/ITS amplicon sequencing"—so I am once again confused. If the authors have shotgun metagenomes, I encourage them to make use of them, to do a more detailed genome-centric analysis of the community members that respond to treatment. Such an analysis would be outside the scope of this work, but there is much more to be learned from whole metagenomes than just the taxonomic distribution of the community.) Second, statements such as "selected an appropriate assembly software" are insufficient, and the authors make no mention at all of binning nor of how they defined OTUs. Third, this paragraph is in a section about DNA sequencing but appears (e.g. ll. 188–191, "Aligned the Clean Reads to the contigs using Bowtie2, counted the number of reads mapped to each contig, and then calculated TPM values with R software. Based on the predicted contig/gene relationships, obtained the expression levels of individual genes") to be describing metatranscriptomic mapping and differential expression analysis. If indeed this is about metatranscriptomics, then we need additional details on RNA extraction, rRNA removal (if any), etc., as the extraction and library prep details in the paragraph above just cover DNA.

Response: We apologize for the confusion caused by our previous response and by the fragmented methods text. We now clarify unambiguously: this study employed shotgun metagenomic sequencing (not 16S/ITS amplicon sequencing and not metatranscriptomics). The Methods section (Section 2.4) has been completely rewritten as coherent paragraphs and now provides full methodological detail.

We appreciate the reviewer’s encouraging suggestion to perform genome-centric analysis of the metagenomes. We fully agree that MAG-resolved analysis would deepen the biological insights, and we have added this as a stated future direction in the Conclusions ("future long-term field studies incorporating multi-omics frameworks"). Such an analysis is beyond the scope of the present study, which focuses on taxonomic community responses and their environmental drivers.

Summary of Additional Improvements

In addition to the point-by-point revisions above, we have:

�Had the entire manuscript professionally edited for English language, grammar, and style, addressing the reviewer’s assessment that the manuscript did not meet PLOS ONE’s standard for intelligible, standard English.

�Ensured consistent and accurate reporting of statistical significance throughout the Results and Discussion sections.

�Corrected the RDA explanatory-power narrative to match the data in Tables 4 and 5.

�Tempered over-strong conclusions regarding combined treatments and microbial network reassembly, and added explicit study limitations and future directions.

�Clarified all sequencing and bioinformatics methods with specific software names, versions, and parameters.

�Redesigned Figures 5 and 6 as LDA bar plots with species-level taxa moved to supplementary materials, and simplified Figures 7 and 8 to genus-level labels only.

We hope that these revisions satisfactorily address all of the reviewer’s concerns and that the manuscript is now suitable for publication in PLOS ONE. We thank the reviewer again for the thorough and constructive feedback, which has significantly improved the quality of our work.

Sincerely,

Na Wang and co-authors

On behalf of all authors

Attachment Submitted filename: Response_to_Reviewers 2.docx

# Editor report: Decision Letter 2

**DOI:** 10.1371/journal.pone.0359576.r005

**Authors:** Massimiliano Cardinale

15 Sep 2026

Soil amendments reshape alpine-meadow microbial communities across degradation gradients by modulating nutrient availability

PONE-D-25-55807R2

Dear Dr. Wang,

We’re pleased to inform you that your manuscript has been judged scientifically suitable for publication and will be formally accepted for publication once it meets all outstanding technical requirements.

Within one week, you’ll receive an e-mail detailing the required amendments. When these have been addressed, you’ll receive a formal acceptance letter and your manuscript will be scheduled for publication.

An invoice will be generated when your article is formally accepted. Please note, if your institution has a publishing partnership with PLOS and your article meets the relevant criteria, all or part of your publication costs will be covered. Please make sure your user information is up-to-date by logging into Editorial Manager at Editorial Manager® and clicking the ‘Update My Information' link at the top of the page. For questions related to billing, please contact billing support.

If your institution or institutions have a press office, please notify them about your upcoming paper to help maximize its impact. If they’ll be preparing press materials, please inform our press team as soon as possible -- no later than 48 hours after receiving the formal acceptance. Your manuscript will remain under strict press embargo until 2 pm Eastern Time on the date of publication. For more information, please contact onepress@plos.org.

Kind regards,

Massimiliano Cardinale, PhD

Academic Editor

PLOS One

Additional Editor Comments (optional):

Reviewers' comments:

# Editor report: Acceptance letter

**DOI:** 10.1371/journal.pone.0359576.r006

**Authors:** Massimiliano Cardinale

PONE-D-25-55807R2

PLOS One

Dear Dr. Du,

I'm pleased to inform you that your manuscript has been deemed suitable for publication in PLOS One. Congratulations! Your manuscript is now being handed over to our production team.

At this stage, our production department will prepare your paper for publication. This includes ensuring the following:

* All references, tables, and figures are properly cited

* All relevant supporting information is included in the manuscript submission,

* There are no issues that prevent the paper from being properly typeset

You will receive further instructions from the production team, including instructions on how to review your proof when it is ready. Please keep in mind that we are working through a large volume of accepted articles, so please give us a few days to review your paper and let you know the next and final steps.

Lastly, if your institution or institutions have a press office, please let them know about your upcoming paper now to help maximize its impact. If they'll be preparing press materials, please inform our press team within the next 48 hours. Your manuscript will remain under strict press embargo until 2 pm Eastern Time on the date of publication. For more information, please contact onepress@plos.org.

You will receive an invoice from PLOS for your publication fee after your manuscript has reached the completed accept phase. If you receive an email requesting payment before acceptance or for any other service, this may be a phishing scheme. Learn how to identify phishing emails and protect your accounts at https://explore.plos.org/phishing.

If we can help with anything else, please email us at customercare@plos.org.

Thank you for submitting your work to PLOS One and supporting open access.

Kind regards,

PLOS One Editorial Office Staff

on behalf of

Dr. Massimiliano Cardinale

Academic Editor

PLOS One
